# Phospho-dependent Regulation of SAMHD1 Oligomerisation Couples Catalysis and Restriction

**DOI:** 10.1371/journal.ppat.1005194

**Published:** 2015-10-02

**Authors:** Laurence H. Arnold, Harriet C. T. Groom, Simone Kunzelmann, David Schwefel, Sarah J. Caswell, Paula Ordonez, Melanie C. Mann, Sabrina Rueschenbaum, David C. Goldstone, Simon Pennell, Steven A. Howell, Jonathan P. Stoye, Michelle Webb, Ian A. Taylor, Kate N. Bishop

**Affiliations:** 1 The Francis Crick Institute, Mill Hill Laboratory, The Ridgeway, Mill Hill, London, United Kingdom; 2 Faculty of Medicine, Imperial College London, London, United Kingdom; 3 Centre for Genomic Medicine, Institute for Human Development, Faculty of Medicine and Human Sciences, University of Manchester, Manchester, United Kingdom; Northwestern University Feinberg School of Medicine, UNITED STATES

## Abstract

SAMHD1 restricts HIV-1 infection of myeloid-lineage and resting CD4^+^ T-cells. Most likely this occurs through deoxynucleoside triphosphate triphosphohydrolase activity that reduces cellular dNTP to a level where reverse transcriptase cannot function, although alternative mechanisms have been proposed recently. Here, we present combined structural and virological data demonstrating that in addition to allosteric activation and triphosphohydrolase activity, restriction correlates with the capacity of SAMHD1 to form “long-lived” enzymatically competent tetramers. Tetramer disruption invariably abolishes restriction but has varied effects on *in vitro* triphosphohydrolase activity. SAMHD1 phosphorylation also ablates restriction and tetramer formation but without affecting triphosphohydrolase steady-state kinetics. However phospho-SAMHD1 is unable to catalyse dNTP turnover under conditions of nucleotide depletion. Based on our findings we propose a model for phosphorylation-dependent regulation of SAMHD1 activity where dephosphorylation switches housekeeping SAMHD1 found in cycling cells to a high-activity stable tetrameric form that depletes and maintains low levels of dNTPs in differentiated cells.

## Author Summary

SAMHD1 is a restriction factor that blocks infection of certain immune cells by HIV-1. It was discovered to be an enzyme that catalyses the breakdown of dNTPs, suggesting that it inhibits HIV-1 replication by reducing cellular dNTP pools to such low levels that reverse transcriptase cannot function. However, recently, alternative mechanisms have been proposed. SAMHD1 is also regulated by phosphorylation, although the effects of phosphorylation on protein function are unclear. In order to address these issues, we carried out combined structural and virological studies and have demonstrated that in addition to allosteric activation and triphosphohydrolase activity, restriction correlates with the capacity of SAMHD1 to form “long-lived” enzymatically competent tetramers. Disrupting the tetramer in various ways always abolished restriction but had differing effects on enzyme activity *in vitro*. SAMHD1 phosphorylation also prevented restriction and tetramer formation but without affecting enzyme catalysis under steady-state dNTP conditions. However phosphorylated SAMHD1 was unable to catalyse dNTP turnover at very low nucleotide levels that more accurately represent conditions in the cells in which restriction takes place. Based on our findings we propose a model for phosphorylation-dependent regulation of SAMHD1 activity and substantiate that degradation of dNTPs by SAMHD1 is sufficient to restrict HIV-1.

## Introduction

HIV-1 replicates poorly in cells of the myeloid lineage and resting T cells through blocks that occur early in infection [1–3]. However, in HIV-2 and related simian viruses (SIVs) the lentiviral accessory protein Vpx overcomes this restriction by targeting the cellular protein SAMHD1 for proteasomal degradation [4–6].

SAMHD1 is a GTP/dGTP-activated deoxynucleotide triphosphohydrolase [7]. It was first identified through association with the rare genetic disorder Aicardi Goutières syndrome (AGS), which mimics congenital viral infection [8]. Human SAMHD1 is a 626 amino acid protein comprising an N-terminal nuclear localisation signal [9], two major structural domains; a sterile alpha motif (SAM) and an HD domain, together with CtD, a C-terminal region required for interaction with Vpx isolated from SIV that infects sooty mangabey (SIV_smm_) [10, 11]. SAM domains are present in a wide range of proteins and are generally involved in protein-protein or protein-nucleic acid interactions [12]. The HD domain contains the active site of the protein and in combination with N- and C-terminal sequences also incorporates the two allosteric nucleotide-binding sites, AL1 and AL2, that regulate the enzyme through combined binding of G-based (AL1) and deoxynucleoside (AL2) triphosphates [7, 13, 14]. The CtD contains the residues bound by SIV_smm_ Vpx that target human SAMHD1 to the ubiquitination machinery and direct its subsequent proteasomal degradation [11].

SAMHD1 is proposed to suppress HIV-1 replication in restrictive cells by inhibiting reverse transcription through depletion of the intracellular dNTP pool. This hypothesis is supported by the inverse relationship between SAMHD1 levels and dNTP concentration (and consequently HIV-1 infectivity) in a number of cell lines and primary cells [15–17]. However, there is uncertainty as to the extent to which the different domains of SAMHD1 contribute to restriction of HIV-1 infection. Some data suggest that only the HD domain is required for inhibition of infection [18] and that removing the C-terminal region does not affect restriction [18, 19], while other studies suggest that the SAM domain may influence function [20] and that the C-terminal region is required for SAMHD1 activity in cells [21]. Although the HD domain has consistently been found to be necessary for lentiviral restriction, some reports have questioned whether this is due to its role in tetramerisation of the protein rather than the presence of the catalytic site [18, 22]. Furthermore, SAMHD1 has been reported to have alternative nuclease [20, 23] and/or RNA binding [24, 25] activities that, dependent on the cellular circumstances, can also mediate restriction of HIV-1 [23]. In addition, restriction is regulated by Threonine 592 phosphorylation [19, 22, 26] and removing this regulation may enable SAMHD1 to inhibit HIV-1 in cycling cells (Cribier et al., 2013).

These latter observations question the role of triphosphohydrolase activity in restriction. However, disparities in the field may be due to variation in assays used to determine enzyme activity or could represent divergent strategies for retroviral restriction. Therefore, given these controversies and the importance of SAMHD1 both as a potential therapeutic target and a key intracellular regulator of metabolism, we set out to thoroughly investigate the activity and regulation of SAMHD1 through a combined virological, structural and biochemical study of the enzyme, both in cells and *in vitro*.

## Results

### SAMHD1 requires both a catalytic site and a C-terminal region for restriction of HIV-1

**Fig 1:**
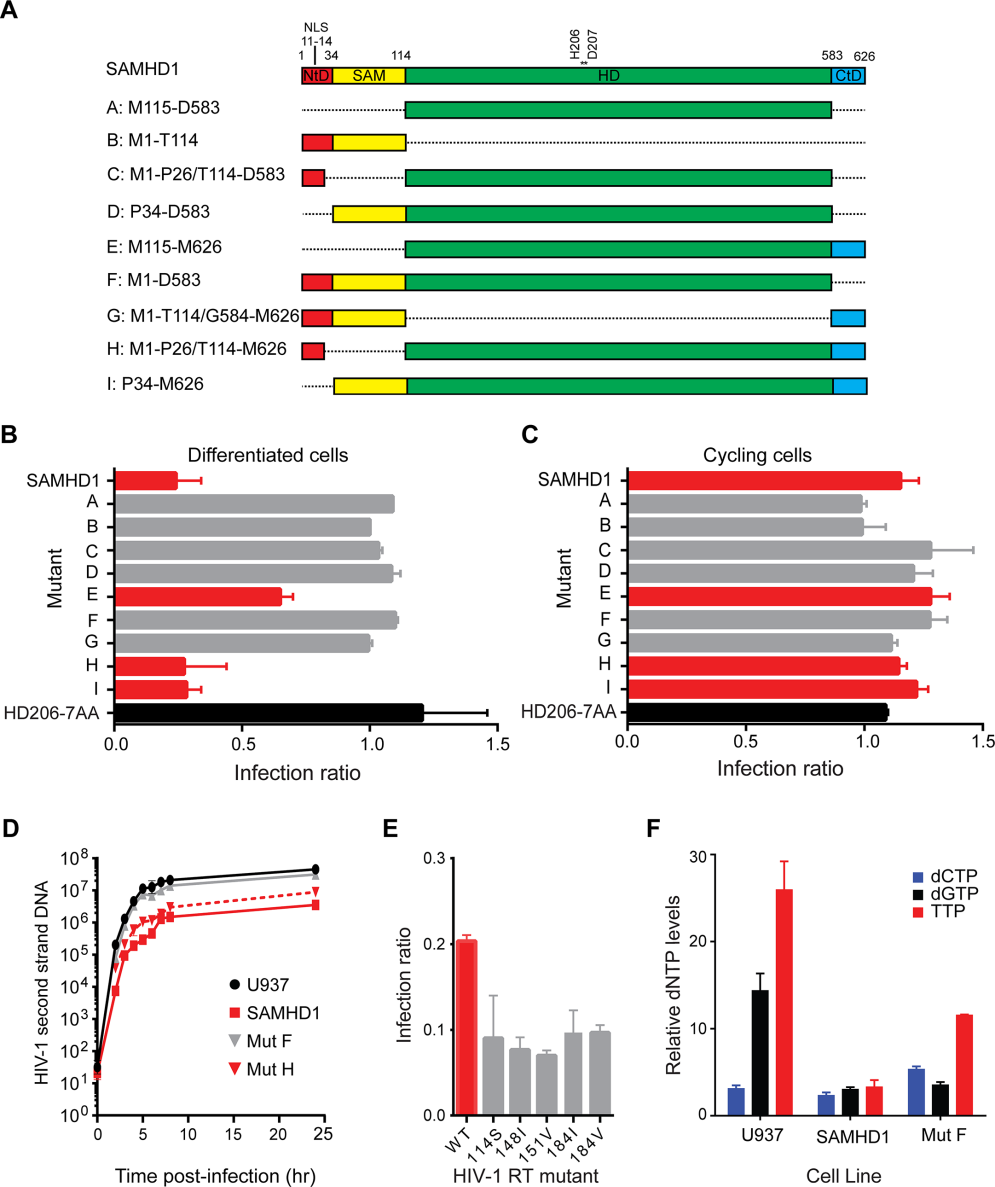
Restriction activity of SAMHD1 deletion constructs. (**A**) Schematic of SAMHD1 mutants showing the N-terminal domain in red with nuclear localisation signal (NLS), the sterile alpha motif (SAM) domain in yellow, the HD domain in green and the C-terminal domain (CtD) in blue. Positions of domain junctions along with active site point mutation residues are numbered above. The identifier (A–I) and amino acids present in each mutant are indicated on the left. (**B**) Restriction of HIV-1 in differentiated cells. U937 cells were transduced with full-length SAMHD1, catalytically inactive SAMHD1(HD206-7AA) or domain mutant VLPs co-expressing YFP. Cells were differentiated after three days and 72 hours later, infected with HIV-1GFP virus. The bar graph shows the degree of restriction compared to SAMHD1 negative cells. Red bars highlight restriction competent SAMHD1. The black bar is the negative control, SAMHD1(HD206-7AA). Error bars represent the range for n ≥ 8 independent experiments. (**C**) Restriction of HIV-1 in in cycling cells. U937 cells were transduced and infected as in **B** but without differentiation. The bar graph shows the degree of restriction with error bars representing the range for n = 3 independent experiments. Red bars represent constructs that are able to restrict HIV-1 in differentiated U937 cells. (**D**) Inhibition of second-strand reverse transcription products by SAMHD1. U937 cells were transduced with SAMHD1 or domain mutant SAMHD1, differentiated and infected with DNase-treated HIV-1GFP. Cells were harvested at the indicated time points post-infection and second-strand reverse transcription products were detected by qPCR. Graph shows a representative experiment (n ≥ 6). Error bars are the standard deviation within the qPCR replicate (**E**) SAMHD1 restriction of HIV-1 RT mutants. U937 cells were transduced with SAMHD1, differentiated as in **B**, and then infected with HIV-1GFP virus carrying the indicated mutations in RT. The bar graph shows relative restriction compared to wild type (WT) HIV-1GFP. Error bars represent the range for n = 3 independent experiments. (**F**) Analysis of cellular dNTP levels. U937 parental cells or cells stably expressing either full-length SAMHD1 or mutant F/ SAMHD(1–583) were differentiated by adding PMA and 72 hours later dNTPs were extracted for quantification. The bar graph shows the relative amounts of each indicated dNTP in each cell line. Error bars represent the range for n = 3 independent experiments.

To determine the regions of SAMHD1 required for restriction activity, a panel of SAMHD1 proteins comprising N- and C- terminal truncations as well as internal deletions was constructed (Fig 1A). The positions at which domains were defined were informed by structural domain boundaries, as they would be more likely to generate stable and active proteins. Expression of each SAMHD1 deletion was assessed by western blotting and only mutants E and H were expressed at lower levels than the full length protein suggesting that most constructs were equally stable in cells (S1 Fig).

Each deletion mutant was assessed for its ability to restrict HIV-1 infection of differentiated U937 cells using a single round, two-colour flow cytometry assay. In this assay, U937 cells were first transduced with a bicistronic IRES vector expressing SAMHD1 and YFP and then differentiated. Three days later, differentiated cells were challenged with HIV-1 virus-like particles (VLP) expressing GFP, and after a further three days, the percentage of YFP-expressing cells that were infected was measured by flow cytometry. As expected, mutants B and G, that did not contain the HD domain, were unable to restrict HIV-1 infection similar to a catalytically inactive mutant, 206-7AA (Fig 1B). However, not all mutants containing the HD domain were able to restrict HIV-1 infection. For example, mutant A, containing only the HD domain, and mutants C, D and F, containing the HD domain in combination with the NLS or SAM domains or both respectively, did not restrict HIV-1 infection (Fig 1B, grey bars). Only mutants E, H and I, containing both the HD domain and the C-terminal domain (CtD, residues 584–626), inhibited infection (Fig 1B, red bars). Therefore, in agreement to previous observations [18, 21] these data demonstrate that both the HD domain and the CtD are required for restriction but that neither the NLS nor SAM domains are necessary. These results were substantiated in stable U937 lines expressing wild type SAMHD1 or mutants F or H (S2 Fig). Restriction by our panel of deletion mutants was also tested in undifferentiated cycling U937 cells using a two-colour assay (Fig 1C). In agreement with previous reports, wild-type SAMHD1 was not able to restrict HIV-1 replication in cycling cells. However, in contrast to a previous observation [19], none of the deletion mutants, including those with a CtD deletion, were able to restrict replication in these cells either.

To test whether the SAM domain influenced the timing of the block to HIV-1 infection, wild type SAMHD1 or domain mutants F (lacking the CtD) or H (lacking the SAM domain) were introduced into U937 cells. Following differentiation and challenge with HIV-1 VLP, the accumulation of reverse transcription products was measured by qPCR (Fig 1D). These data show that wild-type SAMHD1 inhibits the accumulation of second strand reverse transcription products by approximately 10-fold. Moreover, mutant H also inhibited the accumulation of reverse transcripts to a similar degree, whereas the inactive mutant F did not affect reverse transcription. Together, these data indicate that the SAM domain has no influence on the timing or the magnitude of the SAMHD1-mediated block to HIV-1 infection.

We also tested the ability of SAMHD1 to restrict a panel of HIV-1 VLP carrying mutations in reverse transcriptase (RT) that reduce the affinity of the enzyme for dNTPs, and therefore reduce RT processivity [27–29]. None of these mutations had a significant effect on viral infectivity. However, all were found to be more sensitive to SAMHD1 restriction (Fig 1E), implying that the affinity of RT for dNTPs correlates with restriction by SAMHD1. In addition, whilst introduction of SAMHD1 in U937 cells substantially reduced the levels of cellular dNTPs, mutant F, which lacks the CtD, did so but to a lesser extent (Fig 1F). This suggests that mutant F has reduced triphosphohydrolase activity in cells, supporting the proposed correlation between dNTP levels and restriction.

### Oligomeric state and catalytic activity of SAMHD1(115–583)

**Fig 2:**
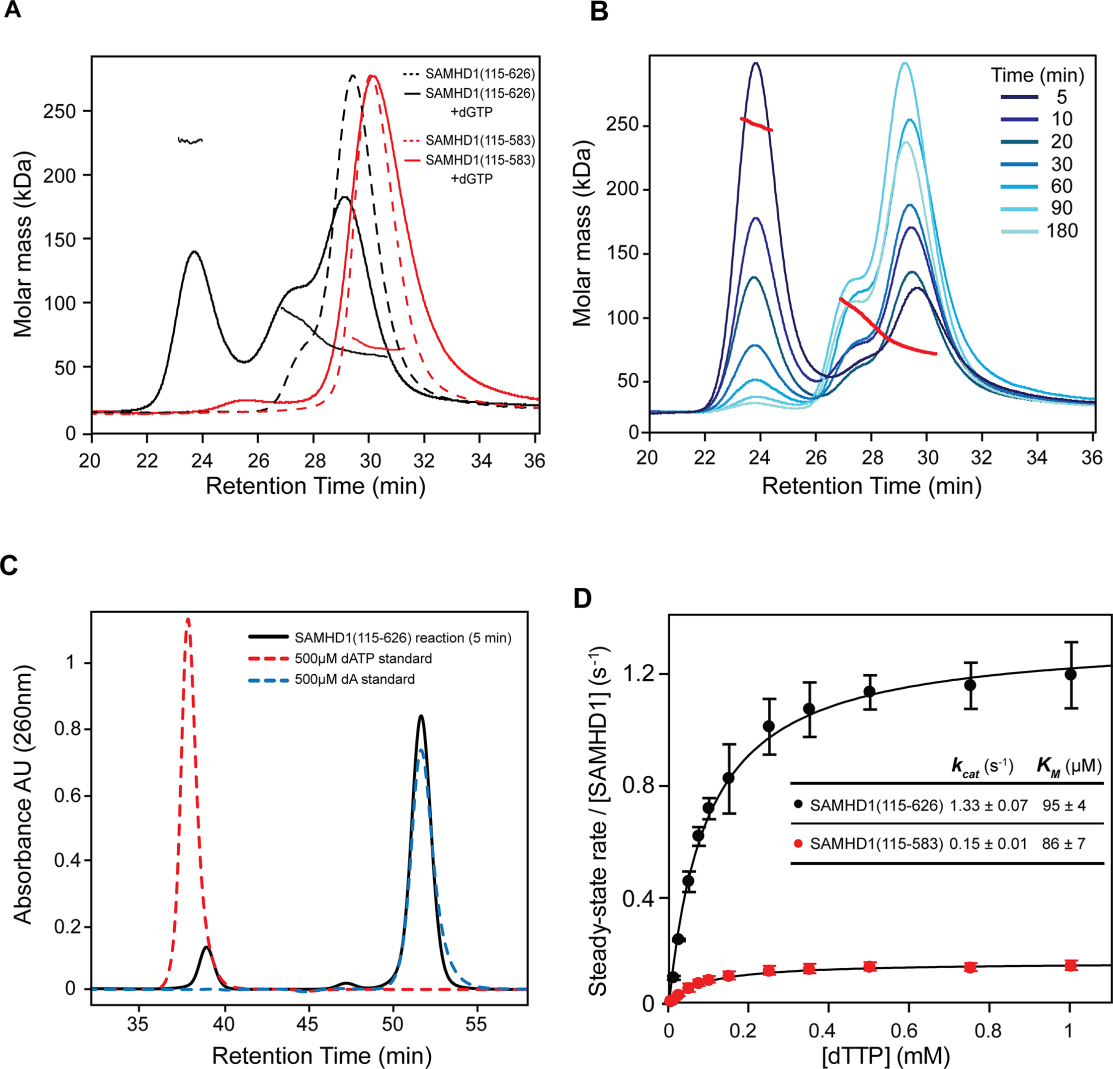
Solution oligomeric state and steady state kinetics of SAMHD1. (**A**) SEC-MALLS analysis of SAMHD1 monomer-dimer-tetramer equilibrium for SAMHD1(115–583) (red) and SAMHD1(115–626) (black). The chromatograms are the output from the differential refractometer. The points are the weight-averaged molar masses determined at 1-second intervals throughout elution of chromatographic peaks. Dashed line chromatograms are apo-protein, solid lines are upon addition of 0.5 mM dGTP (**B**) Tetramer stability. SAMHD1(115–626) was incubated with 0.1 mM GTP and 0.5 mM dATP for the specified time intervals and then the oligomeric state analysed by SEC-MALLS. (**C**) Analysis of dATP hydrolysis during the time course shown in **B**. The chromatogram (black) is the recorded UV absorbance at 260 nm from the SEC-MALLS column for the five-minute incubation time point. Reference chromatograms (dashed lines) for substrate dATP (red) and product dA (blue) are overlaid. (**D**) Steady-state kinetic analysis of GTP stimulated hydrolysis of TTP by SAMHD1. The dependence of the rate of on substrate concentration for SAMHD1(115–626) (black) and SAMHD1(115–583) (red) are plotted. Solid lines are the best fit to the data using the Michaelis-Menten expression. Error bars represent the standard error of the mean (SEM) of three independent measurements. The derived constants K_M_ and k_cat_ for the reaction are displayed inset.

Deletion of the CtD region of SAMHD1, residues 584–626, ablated its HIV-1 restriction activity in differentiated U937 cells. This region has been shown to promote tetramerisation of the protein [13, 14, 21]. Therefore, to examine the effects of this truncation *in vitro* we compared the solution oligomeric state of SAMHD1(115–626) and SAMHD1(115–583) following addition of substrates and allosteric regulators using Size Exclusion Chromatography coupled to Multi-Angle Laser Light Scattering (SEC-MALLS). Addition of the allosteric regulator dGTP to SAMHD1(115–626) resulted in formation of a ~225 kD species corresponding to a protein tetramer (Fig 2A). In contrast, addition of dGTP to SAMHD1(115–583) failed to produce a tetrameric species that was stable under the conditions of the size exclusion chromatography (Fig 2A). Other G-based nucleotides, GTP or ddGTP, were insufficient to induce tetramerisation of SAMHD1(115–626) (S3A Fig). However, when GTP was combined with dNTP substrates that also act as AL2 activators [14, 30, 31], a varying amount of tetramer was formed, dependent on the deoxynucleotide employed (S3B Fig). dATP and dGTP support the greatest degree of tetramer formation, TTP is intermediate and dCTP appears to be ineffective. This same rank order has also been reported for the AL2 dNTP binding preference based on the site occupancy of crystal structures [30]. In addition, the GTP/dATP induced tetramer is highly stable (Fig 2B) as even though more than 90% of the available dATP is hydrolysed to deoxyadenosine (dA) after 5 minutes incubation (Fig 2C), tetramers still persisted for up to 3 hours before the nucleotide-free monomer-dimer equilibrium was re-established. These observations are consistent with chemical crosslinking studies that showed SAMHD1 tetramers persist for hours after dilution of activating nucleotides [31].

Having demonstrated that residues 584–626 are required for stable tetramer formation, a comparison of the steady-state kinetics of dNTP hydrolysis by SAMHD1(115–626) and SAMHD1(115–583) was undertaken using quantitative real-time measurements of triphosphohydrolase activity [32]. In these experiments, GTP was employed as an AL1 allosteric activator as it is not hydrolysed by SAMHD1 and TTP was used as both an AL2 allosteric activator and a substrate. The concentration dependency of the enzyme rate was then fitted to a steady-state Michaelis-Menten model, to derive the kinetic parameters. These data (Fig 2D), show that both enzymes readily hydrolyse TTP, although SAMHD1(115–583) had ~ 10-fold reduced k_cat_ (0.15 sec^-1^ compared to 1.3 sec^-1^). Both enzymes had a similar K_M_ (~90 μM) indicating that the active site is competent and the affinity for substrate/transition state is unchanged. Therefore, although stable tetramer formation is not a prerequisite for SAMHD1 catalysis under steady-state conditions, it does support a higher turnover form of the enzyme and this appears necessary for restriction.

### SAMHD1 tetramer structures

**Fig 3:**
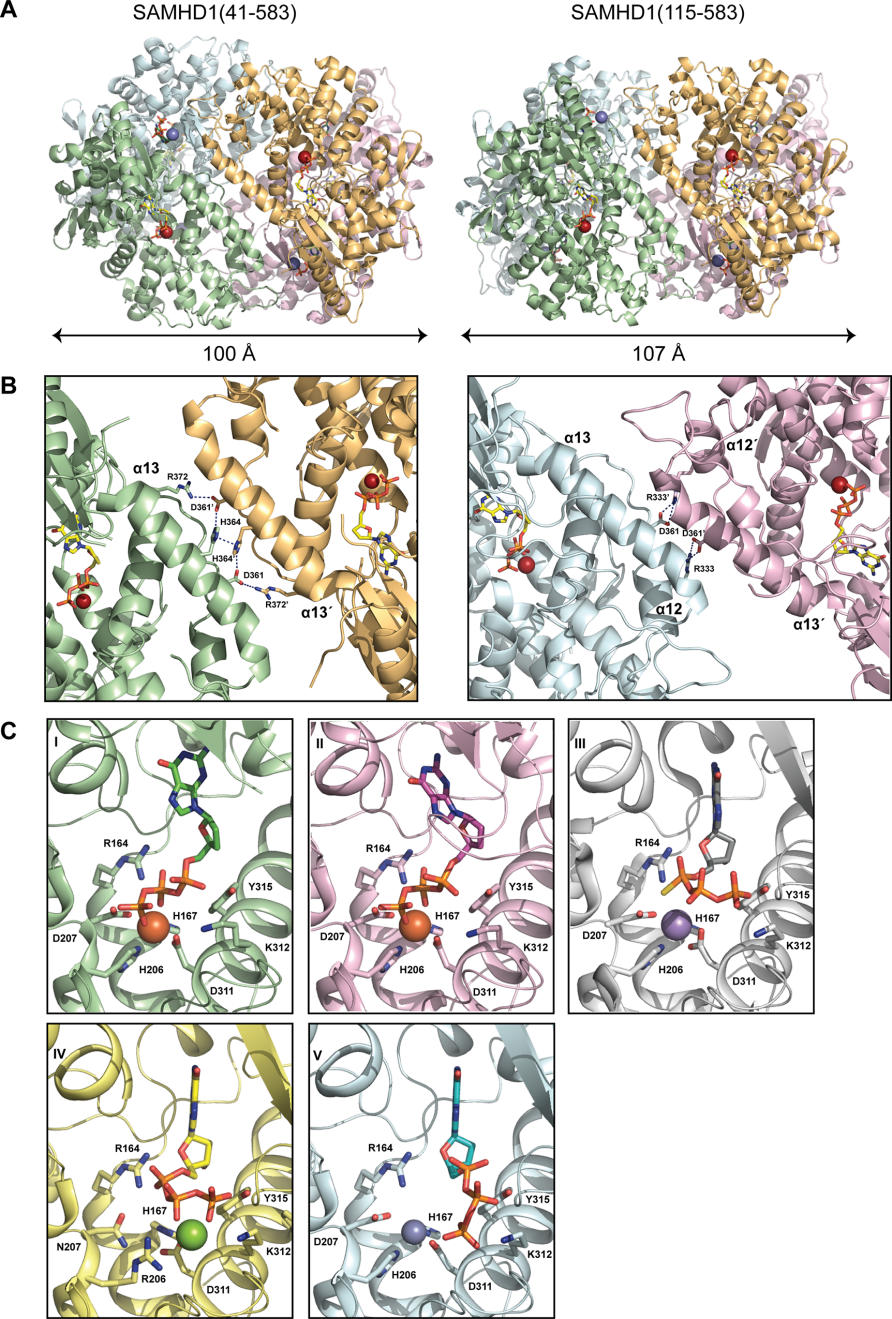
Structures of SAMHD1 tetramers. **(A**) Crystal structures of SAMHD1(41–583) (left) and SAMHD1(115–583) (right) tetramers. Individual monomers are coloured cyan, green, magenta and orange. Bound ddGTP molecules in the active and allosteric sites are shown in yellow and green stick representation respectively. Metal ions are shown as spheres. (**B**) The conserved dimer-dimer interface of SAMHD1(41–583) and SAMHD1(115–583) (left) and the non-conserved SAMHD1(115–583) dimer-dimer interface (right). For clarity only two SAMHD1 monomers, comprising one dimer-dimer interface are shown in each panel. Active site bound ddGTP and residues that make interactions at the subunit interface are shown as sticks. Dashed lines represent hydrogen bonding interactions. (**C**) A comparison of the bound nucleotide conformation and metal configuration of SAMHD1 tetramers is shown. Nucleotides are shown in stick representation, metal ions as spheres: Fe (brown), Mn (purple), Mg (green) and Zn (blue). SAMHD1 residues that chelate metal ions and/or triphosphate are labelled. (I) SAMHD1(41–583)-ddGTP, (II) SAMHD1(115–583)-ddGTP, (III) SAMHD1(113–626)-dGTPαS [13] (PDB ID 4BZC), (IV) SAMHD1(113–626 HD206/207RN)-dGTP [13] (PDB ID 4BZB), (V) SAMHD1(109–626)-dGTP [14] (PDB ID 4MZ7).

To identify residues important for substrate binding, catalysis and tetramerisation we determined the co-crystal structures of SAMHD1(41–583) and SAMHD1(115–583) with the poorly hydrolysable G-based nucleotide analogue, dideoxyguanosine triphosphate (ddGTP). Details of the data collection, structure solution and refinement are presented in Table 1. SAMHD1(41–583) contains the SAM domain however no electron density for the domain was observed, suggesting a degree of flexibly in the linkage between the SAM and HD domain. The CtD is absent in SAMHD1(41–583) and SAMHD1(115–583), and only the AL1 activator ddGTP is present, nevertheless, although not stable under the conditions of our MALLS experiments, both structures are tetrameric, made up from a dimer of dimers (Fig 3A). The primary dimer interface is the same as that observed in other ligand-bound SAMHD1 tetramers [13, 14] and in the apo-stucture [7]. However, the conformation of the tetramer differs, largely due to how the α13 helices at the dimer-dimer interfaces pack against each other. The SAMHD1(41–583) tetramer is symmetrical containing two equivalent dimer-dimer interfaces. In each interface, interactions between R372, D361 and H364 form a hydrogen-bonding network along the whole length of α13 (Figs 3B, left, and S4A). The same α13-mediated dimer-dimer interaction is also observed in the SAMHD1(115–583) but in this case, only in one of the dimer-dimer interfaces. In the second interface, only the first three N-terminal turns of α13 pack against each other (Figs 3B, right, and S4A) and the side chain of D361 makes an alternative hydrogen bonding interaction with R333 located on α12' of the opposing monomer. This non-equivalence results in a SAMHD1(115–583) tetramer with an asymmetric arrangement and importantly also reveals that SAMHD1 tetramers have a degree of conformational flexibility.

**Table 1:** X-ray data collection and structure refinement statistics.

	SAMHD1(41–583) ddGTP	SAMHD1(115–583) ddGTP	SAMHD(115–583) R164A dGTP	SAMHD1(115–626) d4T, GTP	pSAMHD1(115–626) GTP, dATP
**Data collection**					
Wavelength (Å)	0.9795	1.735	0.92	0.9795	0.9795
Space group	R32	P2_1_	P2_1_	P1	C2
Cell dimensions					
*a*, *b*, *c* (Å)	163.2, 163.2, 260.7	70.1, 187.4, 81.4	69.7, 199.3, 81.8	81.9, 95.7, 97.6	197.55, 80.82, 147.66
α, β, γ (°)	90.0, 90.0, 120.0	90.0, 100.5, 90.0	90.0, 100.8, 90.0	91.4, 109.0, 115.3	90.0, 114.9, 90.0
Resolution (Å)	50.00 (3.96)–3.73	50.00 (2.70)–2.54	30.00 (3.14)-2.97	30.00 (3.18)-3.00	30.00 (3.92)-3.70
*R*_sym_ or *R*_merge_ (%)	17.0 (58.8)	7.6 (79.7)	11.5 (74.1)	5.8 (40.7)	10.8 (42.4)
*I* / σ(*I)*	12.23 (4.37)	17.96 (1.61)	13.06 (1.96)	10.52 (1.9)	9.76 (3.23)
Completeness (%)	98.7 (92.5)	96.6 (80.0)	95.9 (81.3)	90.9 (83.1)	95.7 (92.8)
Redundancy	9.67 (8.26)	6.26 (4.12)	3.45 (3.13)	1.73 (1.69)	2.85 (2.75)
**Refinement**					
Resolution (Å)	50.00–3.73	50.00–2.54	30.00–2.97	30.00–3.00	50.00–3.7
No. reflections	13997	65181	43459	45128	22017
*R*_work_ / *R*_free_	21.88 (26.77)	17.09 (22.01)	15.62 (23.44)	17.71 (24.73)	24.39 (30.82)
No. atoms					
Protein	6785	14733	13993	14396	12094
Nucleotide	120	240	124	128	128
Ligand Fe	2	4	4	4	4
Ligand Mg	2	4	-	-	-
Ligand SO4	-	20	-	55	-
Water	3	170	4	5	-
*B*-factors					
Protein	93.4	64.0	64.8	64.8	76.3
Nucleotide	96.2	81.3	85.4	109.9	52.9
Ligand Fe	60.9	45.4	62.9	32.7	50.0
Ligand Mg	79.3	82.2	-	-	-
Ligand SO4	-	140.7	-	119.4	-
Water	70.6	50.7	48.3	29.6	-
R.m.s. deviations					
Bond lengths (Å)	0.006	0.011	0.011	0.01	0.005
Bond angles (°)	1.056	1.532	1.501	1.338	1.050
PDB Code	5ao0	5ao1	5a02	5ao3	5ao4

*Values in parentheses are for highest-resolution shell

### SAMHD1 active sites

In SAMHD1(41–583) and SAMHD1(115–583) the active site contains bound ddGTP and similar to previously described SAMHD1 structures [7, 13, 14], a tightly bound metal ion is co-ordinated by the HD motifs (H167, D311 and H206, D207) and the triphosphate moiety of the bound deoxynucleotide. However, comparison with other SAMHD1 tetramer structures crystallised in the presence of a variety of metals [13, 14] reveals both differences in the nucleotide position and also in the location of the metal centre (Figs 3C and S4B). Specifically, these differences affect how residues R164 and D207 interact with the triphosphate and also how the α, β and γ phosphates of the nucleotide are positioned with respect to the metal ion. Given these variations in active site configuration, the nature of the bound metal was examined by recording anomalous diffraction data from a SAMHD1(115–583)-ddGTP crystal at the Fe absorbance edge and by measuring the X-ray fluorescence emission. Inspection of the anomalous-difference electron density (S5A Fig) readily identifies a first series transition metal, attributed to Fe. However, the X-ray fluorescence emission spectrum (S5B Fig) contains peaks at the Zn and Fe emission energies indicating the presence of both elements, findings also confirmed using inductively coupled plasma mass spectrometry (ICP-MS) (S5C Fig). These data reveal the presence of Fe and Zn, in a variety of different SAMHD1 constructs, albeit at varying ratio but with a total stoichiometry of approximately 1:1 metal to protein indicating that both Zn and Fe can occupy the active site. Taken together, these data demonstrate that the SAMHD1 active site can accommodate different metal ions and that the configuration of bound nucleotides is dependent on the type of metal. Moreover, given that Zn is strongly inhibitory to SAMHD1 triphosphohydrolase activity (S5D Fig) this supports the notion that differential metal incorporation by SAMHD1 might be a means to regulate SAMHD1 activity and substrate selectivity.

### G-based nucleotides in the allosteric site

In the tetramer structures, as well as occupying the active site, ddGTP was also present at the four allosteric AL1 sites, located at the conserved dimer interfaces. To ascertain if a differential preference for G-based nucleotides in the allosteric site exists, two further structures were determined, one with dGTP included in the crystallisation of an active site mutant SAMHD1(115–583, R164A) and the other with GTP and the nucleotide analogue inhibitor 2′,3′-didehydro-2′,3′-dideoxythymidine (d4T) included in the crystallisation of a SAMHD1(115–626) construct. Details of the data collection, structure solution and refinement are presented in Table 1. Both of these crystal forms also contain SAMHD1 tetramers. The SAMHD1(115–583, R164A) structure has the same asymmetric conformation observed in the SAMHD1(115–583)-ddGTP structure and has dGTP bound at the AL1 allosteric site. Nucleotides are absent from the active site likely as a result of the introduction of the R164A substrate binding mutation. SAMHD1(115–626) was crystallised in the presence of both GTP and d4T. The asymmetric unit again comprises four SAMHD1(115–626) molecules arranged as two dimers but with only a minimal contact between one pair of adjacent α13 helices. In this structure, GTP occupies AL1 and again the active site is empty.

**Fig 4:**
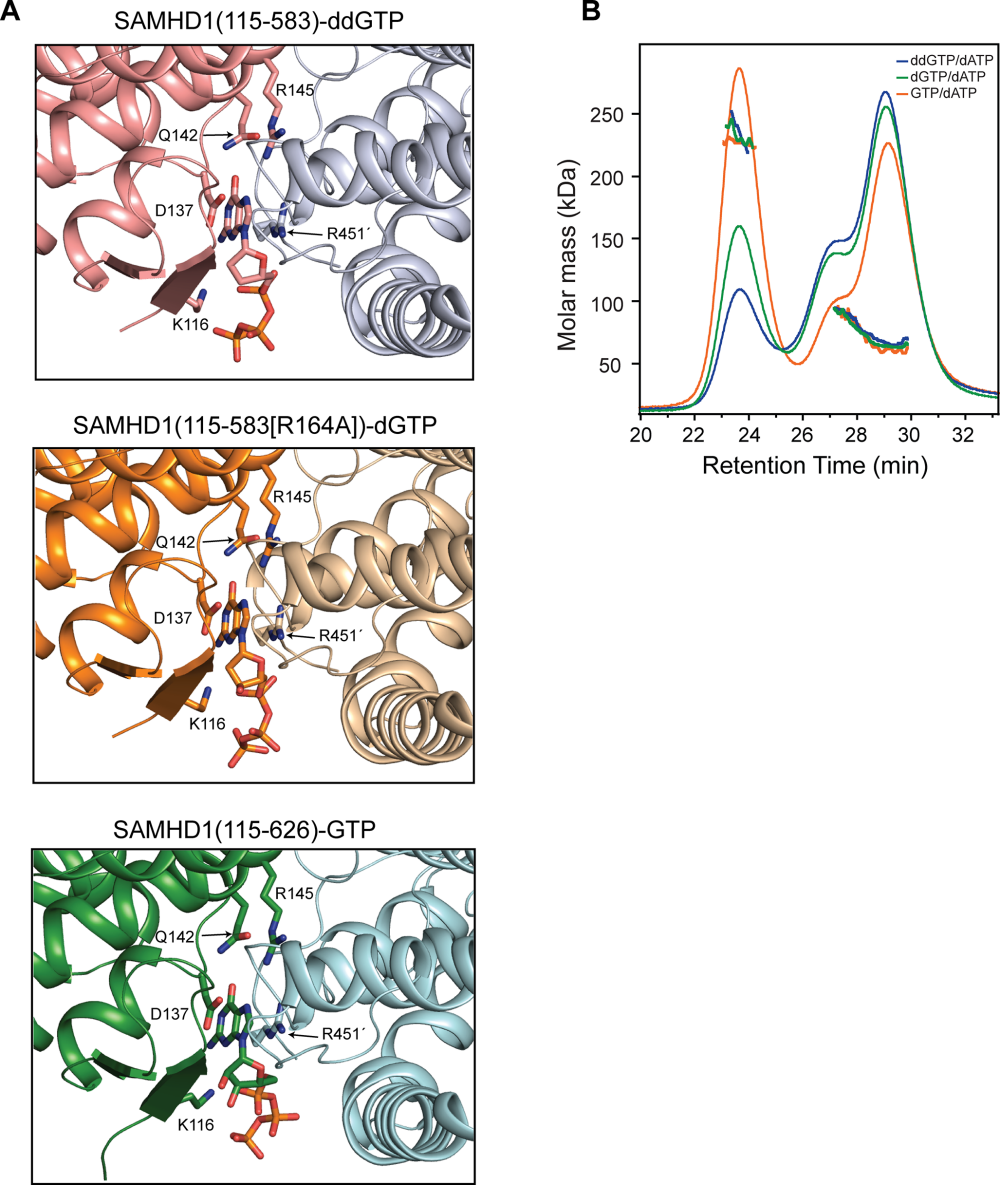
Different G based nucleotides can be accommodated in the SAMHD1 allosteric site. (**A**) The contents and conformation of the allosteric sites for structures of SAMHD1(115–583)-ddGTP (top), SAMHD1(115–583[R164A])-dGTP (middle) and SAMHD1(115–626)-GTP (bottom) are shown. Nucleotides are shown in stick representation, SAMHD1 residues making contacts with the nucleotides are labelled. (**B**) SEC-MALLS analysis of SAMHD1(115–626) incubated with ddGTP/dATP(blue), dGTP/dATP (green) or GTP/dATP (orange). The chromatograms are the output from the differential refractometer. The points are the weight-averaged molar masses determined at 1-second intervals throughout elution of chromatographic peaks.

A comparison of the nucleotide configuration at the AL1 site for ddGTP, dGTP and GTP bound structures is shown in Fig 4A. The electron density that nucleotides were built into is presented in S6 Fig. The G base in all three structures makes the same hydrogen bonds with D137, Q142 and R145 of one monomer along with a stacking interaction with R451' of the opposing monomer. The basic side chains of K116 and R451' also make equivalent electrostatic interactions with the triphosphate of the bound nucleotide illustrating that the binding mode for each of the guanine nucleotides is broadly conserved. Therefore, the capacity of each G-based nucleotide to support tetramerisation when combined with dATP was assessed using SEC-MALLS (Fig 4B). These data show that all G-based nucleotides facilitate tetramer formation but to differing degrees in a rank order of GTP > dGTP > ddGTP. This supports the notion that in a cellular context, where the concentration of GTP is greater than that of any dNTP counterpart, tetramer formation is not limited by the availability of guanine-based nucleotides to occupy AL1, but instead by the availability and capacity of dNTPs to productively support tetramer formation through occupancy of AL2.

### Residues that mediate dNTP hydrolysis, allostery and tetramerisation, are all required for SAMHD1 restriction activity

**Fig 5:**
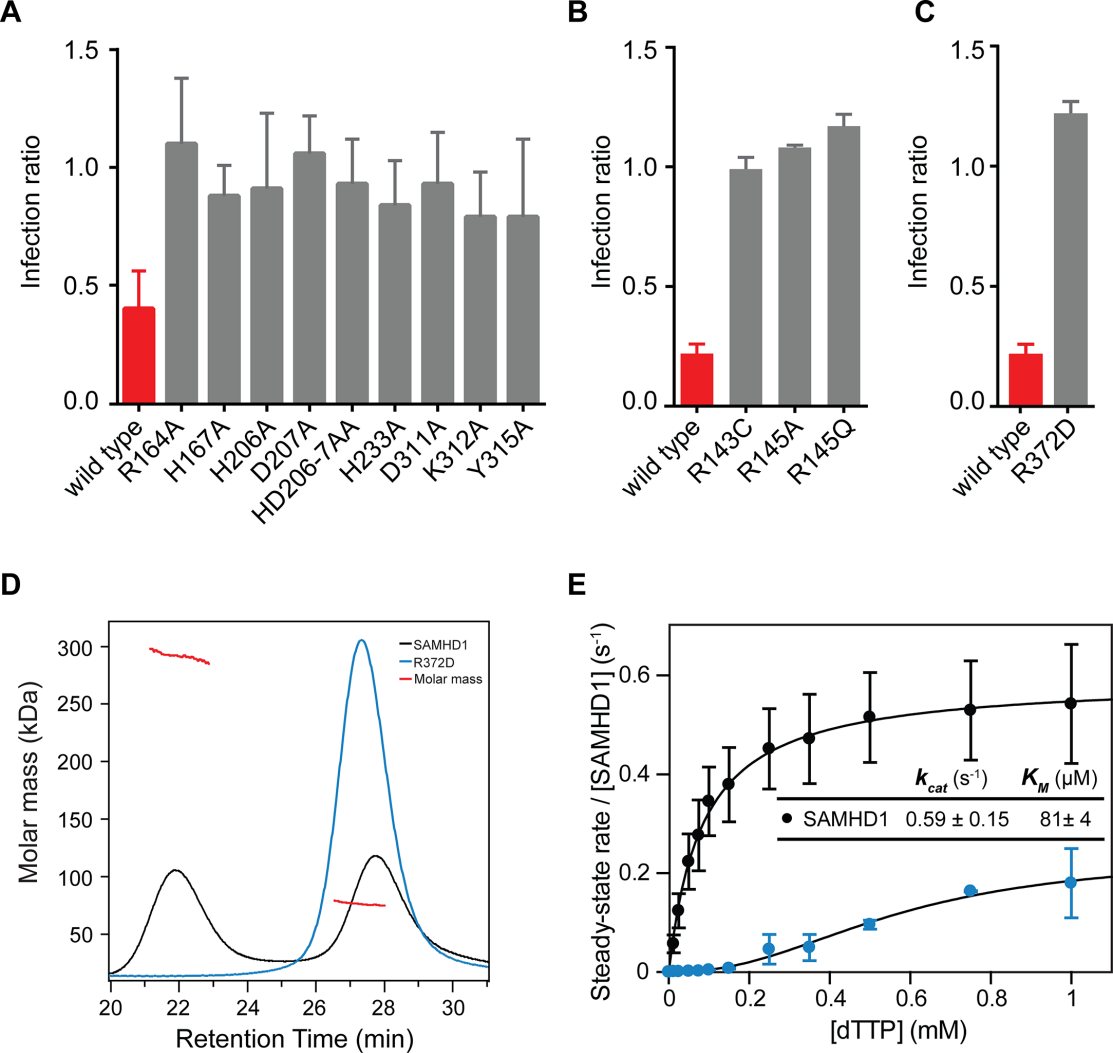
Restriction by active site, allosteric site and tetramer mutants. U937 cells were transduced with the indicated SAMHD1 point mutations at (**A**) the active site, (**B**) the allosteric site or (**C**) the tetramer interface. Cells were differentiated, infected and analysed as in Fig 1B. The bar charts show the fold restriction. The error bars represent standard deviation of the mean (n≥3). (**D**) SEC-MALLS analysis of wild type SAMHD1(black) and SAMHD1(R372D) (blue) incubated with 0.1 mM GTP and 0.5 mM dATP. The chromatograms are the output from the differential refractometer. The red points are the weight-averaged molar masses determined for the two peaks in wt. SAMHD1. (**E**) Steady-state kinetic analysis of GTP stimulated hydrolysis of TTP, by SAMHD1 (black) and SAMHD1(R372D) (blue). The dependence of the rate of on substrate concentration is plotted. Error bars represent SEM of two independent measurements. Solid lines are the best fit to the data using the Michaelis-Menten expression. The derived constants K_M_ and k_cat_ for wt. SAMHD1 are displayed inset.

Based upon our observations in the crystal structures, amino acid substitutions were made to disrupt key residues in the active site of the HD domain, the allosteric site and at the dimer-dimer interface. The SAMHD1 mutant proteins were then tested for inhibition of HIV-1 infection using the two-colour restriction assay (Fig 5). These data show that when residues that co-ordinate the bound iron in the active site (H167, H206, D207 and D311) were replaced with alanine, restriction activity was abolished, confirming that SAMHD1 metal co-ordination in the active site is a requirement for restriction. In addition, alanine substitution of residues that make hydrogen bonds with the phosphates of the bound nucleotide (R164, H233, K321 and Y315) also abolished restriction activity highlighting the necessity for dNTP substrate binding for restriction activity (Fig 5A). Further alanine substitutions were introduced at allosteric site residues R143 and R145 that are mutated in AGS patients [8]. R145 is involved in guanine base recognition and R143 projects from the allosteric site to the rear of the active site. These alanine mutations also abolished SAMHD1 restriction activity (Fig 5B), demonstrating that allosteric activation of SAMHD1 is a further requirement for restriction.

Residue R372 is located at dimer-dimer interface of the tetramer structures and contributes substantially to the hydrogen-bonding network between the adjacent α13 helices (Fig 3B). Therefore, we examined the effects of a charge reversal mutation, R372D, on SAMHD1 restriction and found that this abolished restriction activity (Fig 5C). Moreover, the R372D mutation completely removed the ability of SAMHD1 to form tetramers (Fig 5D) and resulted in a loss of SAMHD1 *in vitro* triphosphohydrolase activity (Fig 5E) supporting the notion that SAMHD1 tetramerisation mediated through allosteric activation is a requirement for both SAMHD1 restriction and dNTP hydrolysis.

### Phosphorylation disassembles the SAMHD1 tetramer to down regulate triphosphohydrolase activity

**Fig 6:**
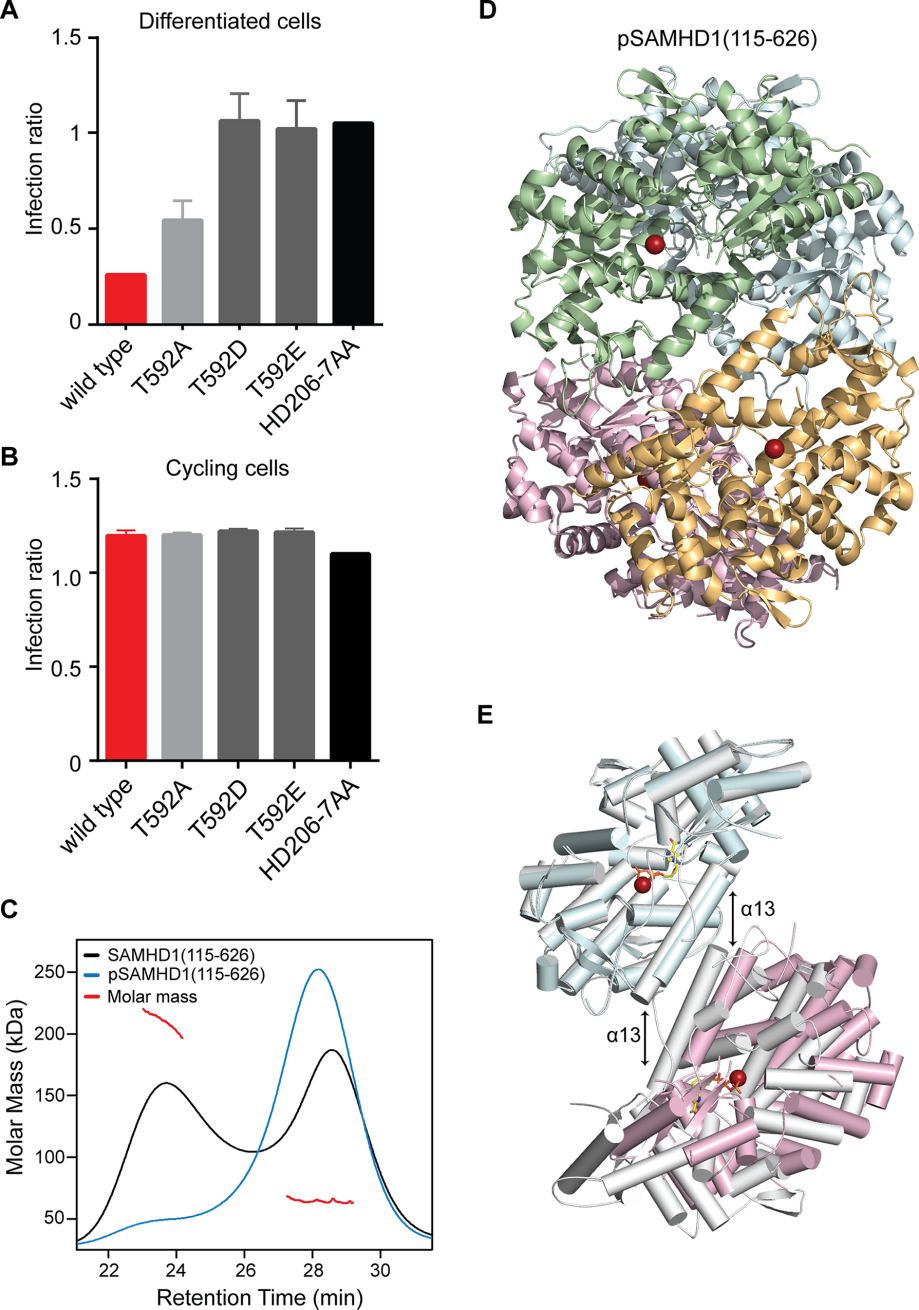
The effect of phosphorylation on SAMHD1 restriction and tetramerisation. Restriction of HIV-1 infection by SAMHD1 Thr592 mutants in (**A**) differentiated and (**B**) cycling U937 cells. Cells were transduced with the indicated SAMHD1 phosphomimetic mutants, differentiated (**A** only), infected and analysed as in Fig 1. Bar charts show the fold restriction. Error bars represent standard deviation of the mean, (n = 6) for **A** and (n = 3) for **B**. (**C**) SEC-MALLS analysis of wild type SAMHD1(115–626) (black) and phospho-SAMHD1(115–626) (blue) incubated with 0.1 mM GTP and 0.5 mM dATP. The chromatograms are the output from the differential refractometer. The red points are the weight-averaged molar masses determined for the two peaks in the wild type protein. (**D**) Crystal structure of pSAMHD1(115–626) tetramer. Individual monomers are coloured cyan, green, magenta and orange. Active site bound Fe metal ions are shown as spheres. (**E**) Structural alignment of pSAMHD1(115–626) (cyan and magenta) and SAMHD1(115–583) (grey) tetramers. For clarity only two SAMHD1 monomers from each structure, comprising one dimer-dimer interface are shown. The structures were aligned on the upper dimer (cyan onto grey) to demonstrate the small displacement in the pSAMHD1(115–626) dimer-dimer interface with respect to that of the SAMHD1(115–583) structure (magenta onto grey). The α13 helix-pairs at the dimer-dimer interfaces are indicated by the double-arrows (left) SAMHD1(115–583) and (right) pSAMHD1(115–626). The active site Fe and ddGTP in SAMHD1(115–583) are also shown in sphere and in stick representation respectively.

Phosphorylation by CDK2/cyclin A or substitution of T592 with phosphomimetic residues has been shown to prevent HIV-1 restriction by SAMHD1 in cycling and differentiated cells respectively. However, how this affects enzyme activity and dNTP levels is uncertain [19, 22, 26]. Our data show that in differentiated U937 cells, alanine substitution at T592 had only a small effect on restriction but that phosphomimetic substitution by aspartate and glutamate completely eliminated the ability of SAMHD1 to restrict (Fig 6A). However, in contrast to Cribier et al. [19], preventing phosphorylation in cycling U937 cells, by, in our case introducing a T592A alanine mutation, did not rescue SAMHD1 restriction (Fig 6B), suggesting that under these circumstances, inhibiting phosphorylation is insufficient to gain restriction.

Given the requirement of the (584–626) CtD region of SAMHD1 for stable tetramer formation *in vitro* and the effects of phosphomimetic and alanine mutations on restriction, the contribution of CDK2/cyclin A phosphorylation on SAMHD1 tetramerisation, and triphosphohydrolase activity were investigated *in vitro*. For these studies SAMHD1 (115–626) was first *in vitro* phosphorylated by incubation with CDK2/cyclin A kinase. After treatment, analysis by tryptic digest and tandem mass-spectrometry found only a single site of phosphorylation at T592 (S7A Fig). In addition, the proportion of phosphorylation was assessed using Phos-tag SDS-PAGE showing that only a single new species was produced and that greater than 90% of the SAMHD1 was modified (S7B Fig) demonstrating that specific and near stoichiometric phosphorylation of SAMHD1(115–626) at T592 was attained.

The capacity for phospho (p) SAMHD1(115–626) to tetramerise upon addition of GTP and dATP was then assessed using SEC-MALLS. These data (Fig 6C) show that phosphorylation also resulted in the inhibition of stable tetramer formation, similar to that observed with SAMHD1(115–583) and the dimer interface mutant R372D. Given these observations, we determined the crystal structure of pSAMHD1(115–626). The structure (Fig 6D) is also tetrameric with the same dimer of dimers conformation mediated by α13 interactions observed in the SAMHD1(115–583) and SAMHD1(41–583) structures. GTP and dATP were included in the crystallisation condition and GTP occupies AL1 but the active site is empty, likely because of hydrolysis of the dATP. Similar to that observed in the SAMHD1(115–583) tetramer in one of the dimer-dimer interfaces, only the first three N-terminal turns of α13 are in close proximity resulting in a tetramer with an asymmetric arrangement. A structural alignment of pSAMHD1(115–626) and SAMHD1(115–583) tetramers also reveals that although the conformation of dimers are near identical (0.58 Å rmsd of all Cα) a small displacement in the positioning of α13-helices (~3 Å) results in a different rotation of one dimer with respect to other between the two tetramers (Fig 6E). The observation of a further alternative dimer-dimer conformation adopted in pSAMHD1(115–626) tetramer strengthens the notion of a conformational plasticity within the dimer-dimer-interfaces of SAMHD1 tetramers.

Although the pSAMHD1(115–626) tetramer includes the CtD region containing the T592 phosphorylation site, no density is observed for residues C-terminal to D583 suggesting that this region of the protein becomes disordered upon phosphorylation. In addition, the pSAMHD1(115–626) structure is much more “open” than the structures of unphosphorylated “closed” tetramers [13, 14] where residues 582–595 comprise a short CtD helix-turn-helix motif (CtD-HTH). In these closed structures residues Q582 and D585, located in the first helix (582–588) of the motif, make hydrogen bonds with R528 and Q536 in the β7-β8 loop region of an opposing SAMHD1 dimer and provide further tetramer-stabilising interactions additional to the α13-α13 interface. T592 is located on the second helix (591–595) where its hydroxyl side chain is hydrogen bonded to the sidechain of D585 as part of the network of interactions that both stabilise and align the helix-turn-helix motif with the opposing β7-β8 loop. In the pSAMHD1(115–626) tetramer, residues C-terminal to D583 are disordered, the CtD-HTH is not formed and so these additional tetramer stabilising interactions do not occur. Moreover, modelling of phosphorylation of T592 into closed structures introduces substantial clashes of the phosphate group with residues in the CtD-HTH, especially with the sidechains of D585 and P593. This gives rise to the notion that T592 phospho-dependent disruption/disordering of the CtD-HTH results in the loss of the additional tetramer stabilising interactions with the β7-β8 loop and that it is these interactions that are required for stable tetramer formation observed by SEC-MALLS.

**Fig 7:**
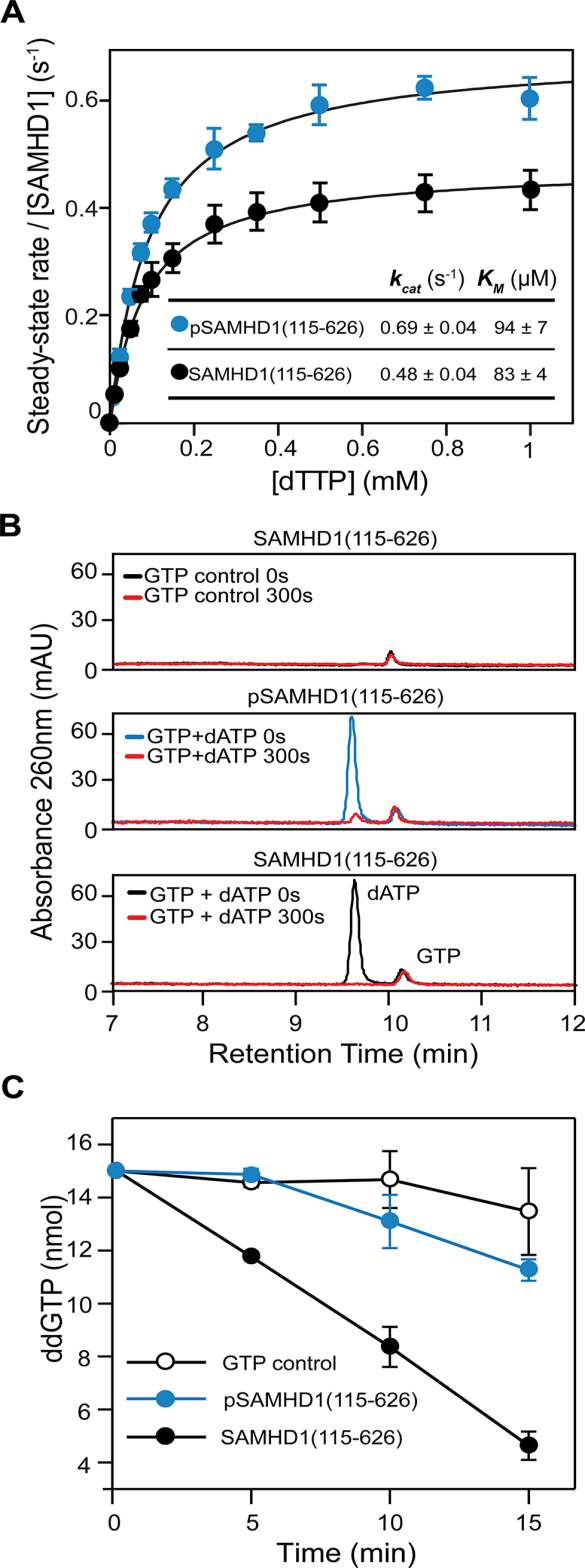
The effect of phosphorylation on SAMHD1 enzyme activity. (**A**) Steady-state kinetic analysis of GTP stimulated hydrolysis of TTP, by SAMHD1 (115–626) (black) and phospho-SAMHD1(115–626) (blue). The dependence of the rate of on substrate concentration is plotted. Error bars represent SEM of three independent measurements. Solid lines are the best fit to the data using the Michaelis-Menten expression. The derived constants K_M_ and k_cat_ are shown inset. (**B and C**) Effect of activator depletion on SAMHD1 and pSAMHD1 triphosphohydrolase activity. (**B**) IEX-HPLC analysis of nucleotide composition after incubation of SAMHD1(115–626) with GTP (upper), and pSAMHD1(115–626) (middle) or SAMHD1(115–626) (lower) with dATP and GTP for 0 and 300 seconds. (**C**) IEX-HPLC analysis of the hydrolysis of ddGTP added to the samples depleted of dATP in (**B**). The plot shows the quantification of the time dependent hydrolysis derived from integration of the ddGTP peak at each time point. Error bars represent the SEM from 3 independent experiments.

To examine how phosphorylation affects SAMHD1 triphosphohydrolase activity we initially employed steady-state kinetics to compare the activity of SAMHD1(115–626) and pSAMHD1(115–626). Surprisingly, these data (Fig 7A) revealed that both phospho- and non-phospho forms have comparable k_cat_ and K_m_ for TTP hydrolysis. However, these experiments were conducted under steady-state conditions at “high” substrate and activator concentration. Therefore, in order to better understand how phosphorylation and tetramer disassembly might regulate restriction, an activator-depletion coupled to an “advantageous” substrate experiment was carried out. Here, SAMHD1(115–626) and pSAMHD1(115–626) were first incubated with GTP and dATP until all substrate dATP was depleted (Fig 7B). Then ddGTP was added and hydrolysis assessed by IEX-HPLC, Fig 7C. ddGTP cannot induce tetramer formation alone (S3A Fig) but unlike other ddNTP substrates can be hydrolysed when other dNTP activators occupy AL2 (S8 Fig). Fig 7C shows that whilst SAMHD1(115–626) readily hydrolyses the newly added substrate pSAMHD1(115–626) is unable to hydrolyse the ddGTP. These data suggest that the capacity to form a stable tetramer allows dNTPs to be retained in the allosteric sites, maintaining the active form of SAMHD1 even when dNTPs are at very low levels. In contrast, phosphorylation of SAMHD1 destabilises tetramer formation resulting in the loss of activating dNTPs and down-regulation of pSAMHD1 activity in a depleted deoxynucleotide environment.

## Discussion

### SAMHD1 triphosphohydrolase activity and restriction

It was the triphosphohydrolase activity of SAMHD1 that was first described to be responsible for restriction of HIV-1 [7, 15]. However, more recently, it has been reported that SAMHD1 has nuclease activity and that this putative RNase activity is its key antiviral property [20, 23]. In addition, there are disputes over which domains of SAMHD1 are required for restriction. Here, we demonstrate that both the HD domain and CtD are required for restriction of HIV-1 in differentiated U937 cells expressing SAMHD1, but that the SAM domain and N-terminal region containing the NLS are dispensable (Fig 1). In contrast to a recent report [20], the presence of the SAM domain does not enhance the magnitude or significantly alter the kinetics of restriction (Fig 1). At the active site, Fe as well as other metals, including Zn, can be co-ordinated (S5 Fig). However, the metal-type affects the configuration of the bound nucleotide (Fig 3C) and Zn actually inhibits triphosphohydrolase activity (S5D Fig), suggesting that differential metal binding at the SAMHD1 active site may have a regulatory function. The importance of nucleotide configuration and metal binding for restriction is also apparent as mutations made at both metal-coordinating residues and those required for nucleotide binding all result in a loss of restriction activity (Fig 5). At the allosteric site, all G-based nucleotides are accommodated and support SAMHD1 triphosphohydrolase activity. Mutation of the AL1 G-recognition residue R145 to alanine or introduction of the AGS mutation R143C that makes hydrogen bonds to both R145 in AL1 and H210 in the active site also both abolish restriction (Fig 5). These data suggest that both binding of the G-based nucleotide in AL1 and the linkage of active and allosteric sites through the R143 “bridge” are requirements for restriction.

These results indicate that inhibition of HIV-1 by SAMHD1 requires the enzyme to be competent for binding both dNTPs and metal ions in the active site and dNTPs at the allosteric site strongly suggesting that restriction is linked to the capacity of SAMHD1 to hydrolyse dNTPs. Further, our data show that HIV-1 with reverse transcriptase (RT) mutations that result in a less processive enzyme or increased K_M_ for deoxynucleotide substrates [27–29] are more sensitive to SAMHD1 (Fig 1E). As these mutants have the same genomic RNA they would be expected to have the same sensitivity to an RNase activity, and so these data further support the notion that the SAMHD1 triphosphohydrolase activity limits dNTP availability for RT and that this inhibits viral infection at the time of reverse transcription.

### Regulation of triphosphohydrolase activity and restriction through tetramerisation and phosphorylation

Our data show that the CtD of SAMHD1 is required for restriction activity (Fig 1). Deletion of the CtD abolishes stable tetramer formation *in vitro* (Fig 2A), although transient tetramers presumably can form as the crystal structures of SAMHD1(1–583) and SAMHD1(41–583) are tetrameric (Fig 3A). Preventing any tetramer formation by mutation of R372, a key residue in the primary α13-α13 tetramer interface, also blocks restriction and severely impairs triphosphohydrolase activity (Fig 5). These data strongly suggest that, in addition to nucleotide binding and hydrolysis, SAMHD1 stable-tetramerisation is also required for restriction. However, although removal of the CtD reduces triphosphohydrolase activity *in vitro*, by around 10-fold, it is not completely abolished, and the K_m_ for substrates is not strongly affected (Fig 2D), indicating that stable tetramerisation is not a mechanistic requirement for catalysis. These data are supported by the observation that the amount of stable tetramer formed is highly dependent on the identity of the nucleotide occupying AL2 (S3B Fig) but regardless, all dNTPs are still hydrolysed at a comparable rate [30]. Again, this suggests that stable tetramerisation is not an absolute requirement for *in vitro* SAMHD1 triphosphohydrolase activity making its role in restriction unexplained.

Recent studies have identified phosphorylation as a means to regulate SAMHD1 activity in cells [19, 22, 26]. However, the mechanism of regulation is unclear, as phosphomimetic mutants display no apparent triphosphohydrolase defect *in vivo* [22, 26]. Moreover, in the reported structures of non-phosphorylated SAMHD1 tetramers [13, 14], the phosphorylation site was remote to both the active and allosteric site of the enzyme. These observations furthered suggestions that SAMHD1 has an alternative activity to dNTP hydrolysis and/or required a co-factor in cells, in order to restrict retroviral replication [22, 26]. Given the degree of conformational flexibility we observe at the tetramer interface (Figs 3 and 6) combined with our *in vitro* tetramerisation data, we questioned whether phosphorylation might regulate tetramer formation. We found that introduction of phosphomimetic mutants at T592 abolishes restriction activity in cells and that phosphorylation of residue T592 *in vitro* inhibits stable tetramer formation (Fig 6C). Moreover, the structure of pSAMHD1 (115–626) has an open conformation highly similar to that of the C-terminally deleted SAMHD1 structures. However, steady-state kinetic measurements of pSAMHD1 revealed that although T592 phosphorylation prevents stable tetramer formation and blocks restriction, it has surprisingly little effect on SAMHD1 dNTP turnover *in vitro* (Fig 7A). This could suggest that triphosphohydrolase activity is not linked to restriction and that tetramerisation is required for an alternative mechanism. SAMHD1 oligomers including tetramers have been observed in cells [21, 25]. However, as the putative RNase activity does not require tetramerisation [23], and our other requirements for restriction are all linked to dNTP binding, this seems unlikely.

Our experiments *in vitro* were performed under steady state conditions that are likely very different to those in non-dividing differentiated myeloid cells and resting T-cells that have low dNTP levels [6, 15–17, 33]. Therefore, we assessed how SAMHD1 activity was affected under conditions of activator-depletion (Fig 7B and 7C) where the enzyme response to the environment may be more important than the steady-state rate of catalysis. By using a ddGTP substrate that is hydrolysed only when a dNTP fills AL2 we were able to separate substrate from activator function (Fig 7C). These data reveal that the inability of pSAMHD1 to form a stable tetramer results in a molecule that is catalytically competent at high nucleotide/activator levels (Fig 7A) but that is unable to hydrolyse substrate at low activator (dNTP) levels (Fig 7C). We therefore conclude that the CtD of SAMHD1 is required to stabilise the tetrameric, active form of the protein by holding it in a closed, tight tetramer form. Phosphorylation of T592 disrupts the CtD, allowing transient, open active tetramers to form but preventing the stabilisation of these tetramers. Removing the CtD also prevents stable tetramer formation and has a more severe effect on the enzyme kinetics. Disruption of the primary α13-α13 dimer-dimer interface has the most severe effect and leads to inability to tetramerise and abolishes triphosphohydrolase activity. Notably, none of our deletion or phosphomimetic SAMHD1 mutants restricted HIV-1 replication in cycling U937 cells (Figs 1C and 6B). Cribier *et al*. reported that a 576–626 SAMHD1 deletion that removes the phosphorylation site enables SAMHD1 to restrict HIV-1 in cycling cells [19]. However, as well as preventing phosphorylation, we would predict that a 576–626 deletion should prevent SAMHD1 stable tetramerisation as observed with our 583–626 CtD mutant (Fig 2A) and therefore would only be functional at high dNTP levels that do not restrict HIV-1 replication.

**Fig 8:**
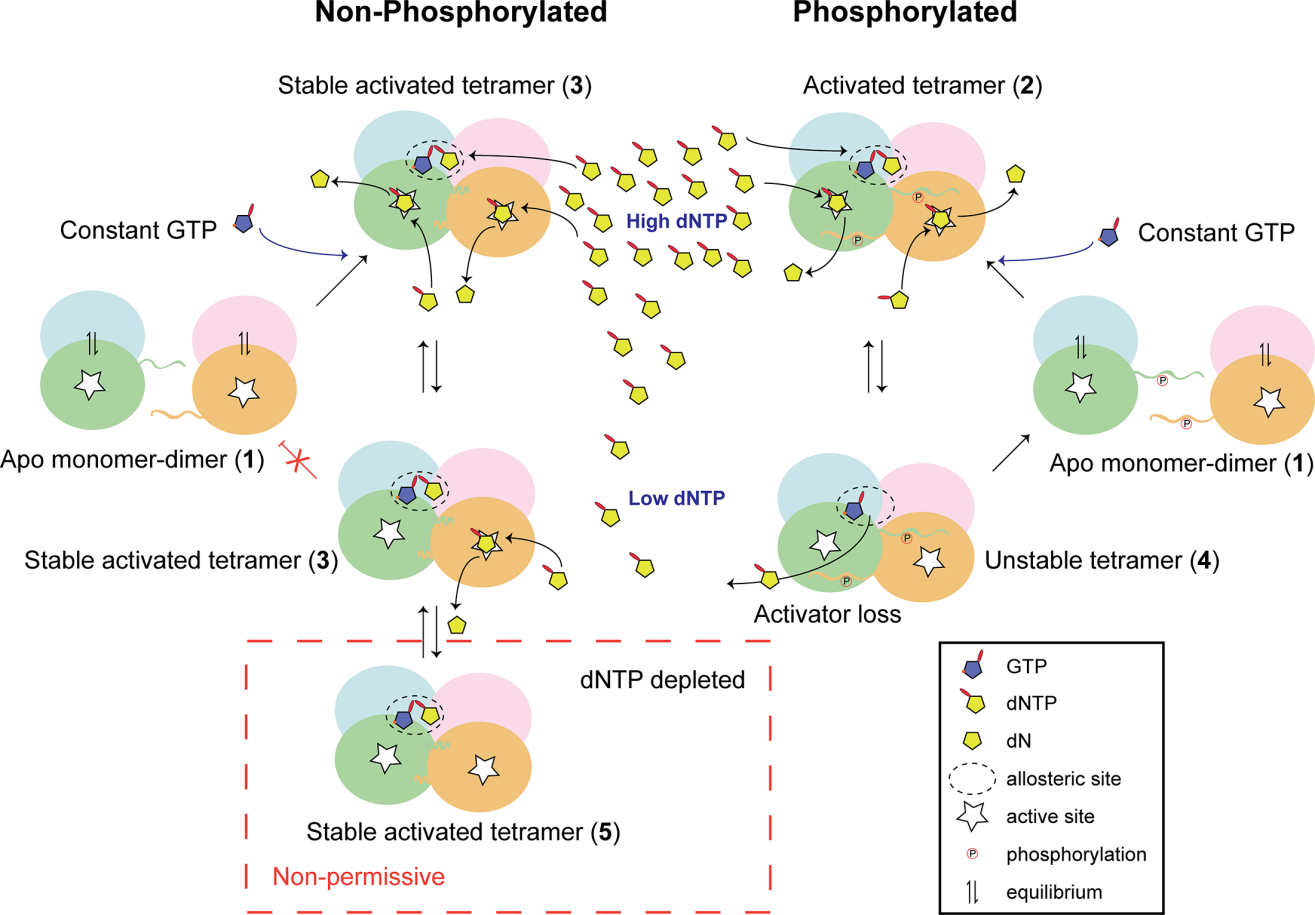
Model for phospho-regulation of SAMHD1 restriction. In the absence of dNTPs Apo-SAMHD1 is found in a monomer-dimer equilibrium regardless of the phosphorylation state (**1**). At high dNTP levels, typically in cycling cells, constitutively abundant GTP combines with dNTPs to fill allosteric sites. In both phosphorylated and un-phosphorylated SAMHD1 this results in the formation of an activated tetramer (**2**) that in the non-phosphorylated protein also includes additional intra-tetramer CtD interactions forming a stable activated tetramer (**3**). Under these conditions, both activated and stable activated tetramers hydrolyse the dNTP pool at comparable rates. At lower dNTP levels, the stabilisation afforded by the CtD interactions maintains enzyme activity in non-phosphorylated SAMHD1 by preventing the loss of dNTP-activator from the allosteric site. However, in phospho-SAMHD1, activating dNTPs dissociate from the allosteric site (**4**) resulting in disassembly of the tetramer and down-regulation of triphosphohydrolase activity. At very low levels, such as in differentiated myeloid cells, CtD-stabilised tetramers still retain activating dNTPs in the allosteric site (**5**) and SAMHD1 remains catalytically competent. It can therefore rapidly respond to any increase in intracellular dNTPs to maintain the dNTP levels below the threshold required for HIV-1 replication.

Given all these observations, a unifying model for SAMHD1 restriction of HIV-1, can be proposed, Fig 8. Here, we postulate that in the differentiated cellular environment depleted of activating dNTPs, only SAMHD1 variants that can form long-lived, stable tetramers are functionally active due to their ability to retain activating nucleotides in the allosteric site [31]. These stable tetramers are necessary and sufficient for restriction of retroviral replication because they can reduce and maintain the dNTP pool to very low levels below the threshold that HIV requires for reverse transcription. By contrast, in cycling cells, dNTP levels are generally higher because of dNTP synthesis through ribonucleotide reductase activity. In these cells, transient pSAMHD1 tetramers can still hydrolyse dNTPs, but as the pool depletes, activating nucleotides can be lost from AL2 and so the enzyme dissociates to inactive dimers and the dNTP pool is never reduced to very low levels. Such low dNTP levels are difficult to accurately measure against background noise in most assays, possibly contributing to conflicting reports in the field. Improved understanding of the mechanism of SAMHD1 activity and its regulation is crucial as, in addition to inhibiting exogenous, and potentially endogenous, viral replication SAMHD1 is an important regulator of intracellular dNTP levels [34]. Our findings therefore have wide-ranging implications in these fields and may also inform the mechanistic pathogenesis of AGS.

## Methods

### SAMHD1 Plasmids and expression

For SAMHD1-YFP expression plasmids, a full-length codon-optimised SAMHD1 sequence was amplified from pLgatewaySN_SAMHD1 [7], sub-cloned into pENTR/D/TOPO using the pENTR Directional Cloning Kit and transferred into pLgatewayIRESEYFP using Gateway LR Clonase II Enzyme mix (Invitrogen). Mutants A, B, D, F and I were amplified from pLgateway_SAMHD1IRESYFP using primers listed in the S1 Table. PCR reactions contained 0.5 μM forward and reverse primers, 200 μM dNTP, 1X Phusion HF buffer, 10 ng template and 1 Unit Phusion High-Fidelity DNA Polymerase (NEB). Cycling conditions consisted of initial denaturation at 98°C for 30 sec, 98°C for 10 sec, 70°C for 30 sec, 72°C for 1 min (for 35 cycles) and final extension of 10 min at 72°C. Inserts were cloned into pLgatewayIRESEYFP as above. Mutants C, G and H were made using Quikchange II Site-directed Mutagenesis Kit (Stratagene) according to the manufacturer’s instructions using pLGateway_SAMHD1IRESYFP as a template for G and H, F for C, H for E and the primers listed in the S1 Table. For puromycin resistance vectors, full-length SAMHD1 and mutant F were amplified from pLgatewaySN_SAMHD1, using primers listed in the S1 Table, and cloned into pCMS28 [35] using restriction digest with XhoI and EcoRI or HpaI, and ligation. Mutant H was created from pCMS28_SAMHD1 as above. Point mutations were created in full-length pLgateway_SAMHD1IRESYFP and Reverse Transcriptase mutations were created in p8.91 [36] using the Quikchange II Site-directed Mutagenesis Kit (Agilent technologies). RT mutant 114S was a gift from S. Okura. SAMHD1 expression in transduced cells was analysed by immunoblotting using 1/500 anti-SAMHD1 3295 (in house) followed by 1/5000 anti-rabbit IRDYE800 (Tebu Bio), and imaged on an Odyssey Infrared Imager (Licor).

### Cells and virus production

293T and TE671 cells were maintained in DMEM (Invitrogen) and U937 cells in RPMI +[L]-Glutamine (GIBCO), each supplemented with 10% heat inactivated foetal calf serum (Biosera) and 1% penicillin/streptomycin (Sigma). U937 cells stably expressing SAMHD1 variants were prepared by transduction with MoMLV-based Puromycin-expressing VLPs, followed by selection with puromycin at 10 μg/mL. MoMLV-based YFP-expressing VLPs were made by cotransfecting 293T cells with pVSV-G (gifted from D. Lindemann), pKB4 [37] and pLGateway_SAMHD1IRESYFP (wild-type or mutants), harvesting 48 hr post-transfection. HIV-1GFP was produced by cotransfection of pVSV-G, p8.91 and pCSGW [38]. MoMLV-based Puromycin-expressing VLPs were made by cotransfection of pKB4, pVSV-G and pCMS28_SAMHD1 (wild-type or mutants). VLPs were titred on TE671 or 293T cells for normalisation prior to infection. Viruses with mutations in Reverse Transcriptase were normalised by HIV-1 p24 ELISA (Perkin Elmer).

### Two-colour flow cytometry

U937 cells (1 x 10^6^) were transduced by spinoculation in a 24 well plate at 1,700 rpm (Sorvall Legend RT 75006441L) for 90 min with 0.5 mL normalised virus in the presence of 10 μg/mL polybrene (to give approximately 30% transduction). Cells were transferred to a 6 well plate and 1.5 mL RPMI added. 3 days later, cells were passaged 1/3, differentiated with 100 nM Phorbol 12-myristate 13-acetate (PMA, Sigma) for 72 hours and then infected with HIV-1GFP (~10 ng CA) in fresh media. For experiments using cycling cells, the differentiation step was omitted. Restriction was assessed after 72 hours by 2-colour flow cytometry using BD LSR II or BDVERSE flow cytometers using single colour controls and automatic compensation [39]. Restriction was calculated by dividing the percentage of transduced (YFP +ve) cells that were infected (GFP +ve) by the percentage of non-transduced cells that were infected. Positive (wild-type SAMHD1) and negative (HD206-7AA catalytic site mutant) controls were included for each experiment. Statistical differences between wild type and mutants were determined using the Mann-Whitney test (Dunn’s multiple comparisons test for reverse transcriptase mutants).

### Quantification of reverse transcription products

U937 cells (4 x 10^6^) were transduced by spinoculation at 1,700 rpm (Sorvall Legend RT 75006441L) for 90 min with 0.5 mL concentrated virus in the presence of 1 μg/mL polybrene (to give >80% transduction). 3 days later, cells were plated at 3.8 x 10^6^ cells per well of a 12 well plate in the presence of 100 nM PMA. After 72 hours cells were chilled at 4°C for 30 min and infected with DNase-treated HIV-GFP (10 ng CA) at 4°C for 30 min and transferred to 37°C (time 0). Cells were harvested at the indicated times post-infection by removing media, washing with ice-cold PBS, trypsinising, resuspending in ice-cold PBS, pelleting at 2000 xg for 2 min at 4°C and flash-freezing on dry ice. DNA was isolated using the DNeasy Blood & Tissue Kit (Qiagen). Eluted DNA was treated with DpnI for 1 hour and quantitation of strong stop and second strand PCR products was determined by qPCR using the primers detailed in S2 Table. PCR reactions contained 9 μM forward and reverse primers, 2.5 μM probe, 1X Taqman Fast Advanced Mastermix and 2 μL DNA. Reactions were carried out in a 7500 Fast Real-Time PCR System (Applied Biosystems) using standard reaction conditions.

### Measurement of deoxynucleoside triphosphate levels in cells

Cellular deoxynucleoside triphosphates were extracted from batches of 4x10^6^ differentiated native and SAMHD1 transduced U937 cells according to [40]. The dNTP levels were quantified by radiolabel incorporation assays performed using oligonucleotide templates detailed in [41] and the procedures described in [42] with the following modifications. Standard curves ranged from 0.05 to 4 pmole, 1 unit of KOD polymerase (Merck Millipore) was used in place of Thermo-Sequenase (GE Healthcare) and 2.5 μM of α-^32^P-dATP was employed as an incorporation label.

### Protein expression and purification

For expression in *E*. *coli* the DNA sequences coding for human SAMHD1 residues M1-M626, M115-M626, P26-M626, M115-D583 and D41-D583 were amplified by PCR and inserted into a pET52b expression vector (Novagen) using ligation independent cloning to produce N-terminal StrepII-tag fusion proteins. The M115-D583 (R164A) and M1-M626 (R372D) mutants were prepared from the parent construct using the Quikchange II kit. All insert sequences were verified by DNA sequencing. Strep-tagged SAMHD1 constructs were expressed in the *E*. *coli* strain Rosetta 2 (DE3) grown in Luria broth at 37°C with shaking. Protein expression was induced by addition of 0.1 mM IPTG to log phase cultures (A_600_ = 0.5) and the cells incubated for a further 20 h at 18°C. Cells were harvested by centrifugation resuspended in 30 ml lysis buffer (50 mM Tris-HCl pH 7.8, 500 mM NaCl, 4 mM MgCl_2_, 0.5 mM TCEP, 1x EDTA-free mini complete protease inhibitors (Roche), 0.1 U/ml Benzonase (Novagen) per pellet of 1 L bacterial culture and lysed by disruption in EmulsiFlex-C5 homogeniser (Avestin). The lysate was cleared by centrifugation for 1 h at 48,000 xg and 4°C then applied to a 10 mL StrepTactin affinity column (IBA) followed by 600 mL of wash buffer (50 mM Tris-HCl pH 7.8, 500 mM NaCl, 4 mM MgCl_2_, 0.5 mM TCEP) at 4°C. Bound proteins were eluted from the column by circulation of 1 mg of 3C protease (GE) in 10 mL of wash buffer over the column in a closed circuit overnight. 3C protease was removed by incubation of the eluent with 500 μL GSH-Sepharose (GE). After centrifugation to remove the resin, the supernatant was concentrated to 5 mL and applied to Superdex 200 16/60 (GE) size exclusion column equilibrated with 10 mM Tris-HCl pH 7.8, 150 mM NaCl, 4 mM MgCl_2_, 0.5 mM TCEP. Peak fractions were concentrated to approximately 20 mg/mL and flash-frozen in liquid nitrogen in small aliquots.

### Crystallisation and structure determination

Prior to crystallisation, protein samples were diluted to 5 mg/mL with gel filtration buffer, supplemented with 1 mM of the appropriate nucleotide (Jena Bioscience). Crystals were produced by hanging drop vapour diffusion at 18°C using an Oryx crystallisation robot (Douglas instruments) and 2 μL droplets containing an equal volume of the protein/nucleotide solution and mother liquor.

Crystals of SAMHD1(41–583)-ddGTP were obtained from hanging drops containing 160 mM succinic acid, 11% PEG 3350, pH 7.0. Crystals of SAMHD1(115–583)-ddGTP were obtained from drops containing 100 mM Bis Tris propane-HCl, 150 mM Na_2_SO_4_, 13.5% PEG 3350 pH 6.5. Crystals of SAMHD1 115–583 R164A-dGTP were obtained from drops containing 0.2 M sodium citrate, 0.1 M Bis Tris propane-HCl, 20% PEG 3350, pH 8.5 and Crystals of SAMHD1(115-626-d4T/GTP) were obtained from drops containing 0.2 M ammonium sulphate and 20% PEG 3350. Crystals of pSAMHD1(115–626) were obtained from drops containing 0.2 M sodium formate and 20% PEG 3350 with 25 mM dATP and GTP included in the crystallisation condition. Details of crystallographic spacegroups, cell dimensions and contents of the asymmetric unit are presented in Table 1.

For data collection, crystals were adjusted to 25% glycerol and flash frozen in liquid nitrogen. Datasets were collected on beamline I04 at the Diamond Light Source, UK at a wavelength of 0.97949 Å or 0.92 Å with the exception of the SAMHD1 115-583-ddGTP dataset, which was collected at a wavelength of 1.735 Å, corresponding to the Fe absorption edge. Data were reduced using the HKL [43] or XDS [44, 45] software suites. Structures were solved by molecular replacement using the program MOLREP [46] implemented in the CCP4 interface [47] using the structure of apo-SAMHD1 120–626 as search model (PDB code 3U1N, [7]).

For the SAMHD1(115–583) crystal, iterative model building with the program Coot [48] combined with positional, real-space, individual B-factor and TLS refinement in phenix.refine [49] produced a final model for SAMHD1 residues 115–276, 282–530, 538–583 (chain A); 115–277, 282–506, 513–530, 547–583 (chain B); 115–276, 284–532, 538–583 (chain C); 115–276, 284–506, 512–530, 547–582 (chain D) with R-/Rfree-factors of 16.49%/22.28%. In the model, 97.98% of residues have backbone dihedral angles in the favoured region of the Ramachandran plot, a further 1.74% are in the allowed regions and 0.28% are outliers.

For the SAMHD1(41–583) crystal, iterative model building with Coot combined with positional, real-space, grouped B-factor, TLS refinement and inclusion of reference restraints from the higher-resolution SAMHD1 115–583 model in phenix.refine produced a final model for SAMHD1 residues 110–276, 285–505, 517–531, 547–582 (chain A); 113–277, 287–463, 466–506, 517–529, 548–583 (chain B) with R-/Rfree-factors of 20.94%/26.71%. In the model, 98.48% of residues have backbone dihedral angles in the favoured region of the Ramachandran plot, 1.28% fall in the allowed regions and 0.23% are outliers.

For the SAMHD1(115–583)-R164A crystal, iterative model building with Coot combined with positional, real-space, individual B-factor and TLS refinement produced a final model for SAMHD1 residues 115–276, 284–505, 515–530, 547–582 (chain A); 115–276, 285–505, 547–582 (chain B); 115–276, 283–483, 490–507, 514–530, 539–542, 547–582 (chain C); 115–276, 285–505, 547–582 (chain D) with R-/Rfree-factors of 15.62/23.44. In the model, 97.8% of residues have backbone dihedral angles in the favoured region of the Ramachandran plot, 1.79% are in the allowed regions and 0.42% are outliers.

For the SAMHD1(115–626 crystal), iterative model building with Coot combined with positional, real-space, grouped B-factor and TLS refinement produced a final model for SAMHD1 residues 115–276, 285–507, 513–530, 547–583 (chain A); 115–276, 285–506, 514–530, 547–583 (chain B); 115–276, 284–506, 515–530, 547–583 (chain C); 115–276, 284–505, 514–530, 547–583 (chain D) with R-/Rfree-factors of 17.71/24.73. In the model, 97.9% of residues have backbone dihedral angles in the favoured region of the Ramachandran plot, 1.97% are in the allowed regions and 0.12% are outliers.

For the pSAMHD1(115–626) crystal, iterative model building with Coot combined with positional, real-space, grouped B-factor and TLS refinement in phenix.refine produced a final model for SAMHD1 residues 115–276, 285–505, 517–526, 547–583 (chain A), 115–276, 285–463, 468–484, 491–505, 516–529, 547–581 (chain B), 115–276, 284–394, 407–460, 468–479, 496–505, 518–522, 547–580 (chain C), 117–273, 286–303, 308–460, 474–484, 500–505, 518–529, 549–553, 560–581 (chain D) with R-/Rfree-factors of 24.39/30.82. In the model, 96% of residues have backbone dihedral angles in the favoured region of the Ramachandran plot, 4% are in the allowed regions and 0% are outliers.

Structural alignment and superposition was undertaken using SSM [50] and LSQMAN [51]. Protein structure figures were prepared using PYMOL [52]. The coordinates and structure factors of SAMHD1(41–583)-ddGTP SAMHD1(115–583)-ddGTP, SAMHD(115–583)-R164A-dGTP, SAMHD1(115–626)-d4T-GTP and pSAMHD1(115–626)-GTP have been deposited in the Protein Data Bank under accession numbers 5ao0, 5ao1, 5ao2, 5ao3 and 5ao4 respectively.

### SEC-MALLS

Size Exclusion Chromatography coupled to Multi-Angle Laser Light Scattering (SEC-MALLS) was used to determine the molar mass composition of SAMHD1 samples upon addition of deoxynucleotide/nucleotide substrates and activators. For the time course assessing tetramer stability, samples were incubated at 4°C for the specified time after the addition of nucleotide substrates and activators. Samples (100 μl) were applied to a Superdex 200 10/300 GL column equilibrated in 20 mM Tris-HCl, 150 mM NaCl, 5 mM MgCl_2_, 0.5 mM TCEP and 3 mM NaN_3_, pH 8.0, at a flow rate of 0.5 ml/min. The scattered light intensity and protein concentration of the column eluate were recorded using a DAWN-HELEOS II laser photometer and an OPTILAB-TrEX differential refractometer (dRI) (d*n*/d*c* = 0.186) respectively. The weight-averaged molecular mass of material contained in chromatographic peaks was determined using the combined data from both detectors in the ASTRA software version 6.1 (Wyatt Technology Corp., Santa Barbara, CA).

### ICP-MS

Inductively coupled plasma mass spectrometry (ICP-MS) was employed to determine the identity of the metal ions bound by SAMHD1. Samples were prepared for ICP-MS by extensive dialysis against 20 mM Tris pH 8.0, 150 mM NaCl, 5 mM MgCl_2_ and 2 mM TCEP and the final protein concentration adjusted to 20 μM. Samples (100 μl) were then added to 3 mL of 2% HNO_3_ and quantitative transition metal analysis carried out using an Agilent 7500cx Inductively Coupled Plasma Mass Spectrometer (Manchester Analytical Geochemistry Unit, University of Manchester). External calibration was accomplished using standards with concentrations of 5, 10, 50, 100 and 200 μg/L. Calibration standards were prepared immediately prior to analysis by dilution of concentrated multi-element stock solutions with 2% sub-distilled HNO_3_ in 18 MΩ deionised water.

### IEX-HPLC assay of SAMHD1 triphosphohydrolase activity

3 μM SAMHD1 was incubated with 0.2 mM GTP activator and 0.5 mM dATP substrate in either Reaction buffer, 20mM Tris-HCl, 150 mM NaCl, 5 mM MgCl_2_, 2 mM TCEP (pH 7.5) or Reaction buffer including 10 μM ZnSO_4_. Reactions were allowed to proceed and samples withdrawn at timed intervals up to 3 minutes and terminated by 10 fold dilution into 18.0% acetonitrile, 25mM Tris-HCl, 1 mM EDTA pH 8.0. For assessment of SAMHD1 hydrolysis of ddNTP substrates 3 μM SAMHD1 was incubated with 0.2 mM GTP/dATP activators together with 1 mM ddNTP substrates. In activator-depletion experiments 3 μM SAMHD1(115–626) or pSAMHD1(115–626) was first incubated with 0.05 mM GTP activator and 0.2 mM dATP in Reaction buffer until all substrate dATP was depleted (5 minutes). After the depletion phase 0.5 mM ddGTP was added and samples of the time-course of hydrolysis withdrawn and terminated as above. The nucleotide hydrolysis reactions were analysed by anion exchange HPLC using a DNA-PAC100 (4 x 50mm) column (Dionex). The column was equilibrated at 30°C at 1 mL/min in 25mM Tris-HCl, 0.5% acetonitrile pH 8.0 (Buffer A). Samples of the reaction (2 nmole) were eluted with a five-minute isocratic phase of Buffer A followed by linear gradient of 0 to 240 mM NH_4_Cl over 12 minutes. Absorbance data from the column eluent was continuously monitored between 200-400nm (2 nm interval) using MD-2010 photodiode array detector (JASCO). Peak integration of the absorbance data recorded at 260 nm was used to quantify the amount of substrate and products at each time point of the reaction.

### Real time measurement of triphosphohydrolase activity

To obtain quantitative, time-resolved information and kinetic parameters for SAMHD1 nucleotide hydrolysis, a coupled assay was employed utilising the biosensor MDCC-PBP [53, 54] to measure phosphate release from combined SAMHD1 triphosphohydrolase and *S*. *cerevisiae* Ppx1 exopolyphosphatase activity in real time, as described previously [32]. In a typical experiment, solutions containing SAMHD1 constructs, Ppx1, MDCC-PBP and GTP were incubated for 5 min in assay buffer (20 mM Tris pH 8.0, 150 mM NaCl, 5 mM MgCl_2_ and 2 mM TCEP) at 25°C before the reaction was initiated by the addition of substrate (TTP). The final concentrations were 100 nM SAMHD1, 10 nM Ppx1, 40 μM MDCC-PBP, 0.2 mM GTP and varying concentration of TTP. The fluorescence intensity was recorded at 430 nm excitation and 465 nm emission over a period of 10–30 min in a Clariostar multiwall plate reader (BMG). Steady state rates were obtained from time courses of P_i_ formation by linear regression of the data points in the linear phase of the reaction. Rates were divided by the SAMHD1 concentration and plotted versus substrate concentration. Apparent dissociation constants for substrate binding (K_M_) and catalytic constants (k_cat_) were then determined by nonlinear least squares fitting using either a hyperbolic or a Hill-function. All measurements were performed in duplicate or triplicate.

### *In vitro* phosphorylation of SAMHD1

SAMHD1 was *in vitro* phosphorylated using preassembled human CDK2/cyclin A complex [55]. In a phosphorylation reaction SAMHD1(115–626) was incubated at a ratio of 75:1 (w/w) with CDK2/cyclin A in 10 mM Tris-HCl pH 7.8, 150 mM NaCl, 4 mM MgCl_2_, 0.5 mM TCEP and 2mM ATP. The reaction was initiated by the addition of SAMHD1(115–626) and incubated for 6 hours at 4°C. The target for phosphorylation was threonine 592 and this was confirmed as the sole site of phosphorylation by in-gel trypsin digestion combined with MALDI-TOF mass spectrometry. The level of phosphorylation was also quantified by applying pre and post-phosphorylated samples to 6% (w/v) acrylamide SDS PAGE gels supplemented with 50 μM Phos-tag [56] and Mn^2+^ (Wako, Japan).

## Supporting Information

S1 Fig Expression of SAMHD1 deletion mutants. U937 cells transduced with SAMHD1 mutant viruses, labelled as in Fig 1A, were harvested at the time of infection with HIV-1GFP and SAMHD1 protein expression analysed in a 4–20% Tris-glycine gel followed by immunoblotting with anti-SAMHD1 antibody. M, marker; un, untransduced U937 cells; FL, full-length SAMHD1; +, SAMHD1-transfected 293T positive cell control. Marker molecular weights are indicated. Molecular weights for each mutant were predicted using the primary amino acid sequence in SeqBuilder (DNAStar) and are shown below along with the percentage YFP positive cells for each transduction.
(TIF)

S2 Fig Restriction activity of SAMHD1 deletions in stable cells lines. U937 cells (Parental) stably expressing either full-length SAMHD1, mutant F or mutant H were differentiated by adding PMA and 72 hours later infected with HIV-1GFP virus. The percentage of GFP positive cells was determined by flow cytometry and plotted relative to the amount of HIV-1GFP virus added to the cells. Error bars represent the range for n ≥7 independent experiments.
(TIF)

S3 Fig Nucleotide stimulated tetramerisation. (**A**) SEC-MALLS analysis of monomer-dimer-tetramer equilibrium for SAMHD1(115–626) upon addition of 0.5 mM GTP (orange), dGTP (green) and ddGTP (blue). (**B**) SEC-MALLS analysis of monomer-dimer-tetramer equilibrium for SAMHD1(115–626) upon addition of 0.2 mM GTP and 0.5 mM each of dATP (red), dGTP (green), TTP (purple) and dCTP (blue). The chromatograms are the output from the differential refractometer and the displayed points are the weight-averaged molar masses determined at 1-second intervals throughout elution of chromatographic peaks. (**C**) SAMHD1 triphosphohydrolase activity against ddNTP substrates. GTP/dATP (0.2 mM ea.) stimulated SAMHD1(115–626) hydrolysis of the indicated ddNTP substrate (1 mM) was analysed by IEX-HPLC. The plot shows the quantification of the time dependant depletion of substrate derived from integration of the ddNTP peak at each time point. Error bars represent the SEM from 3 independent experiments.
(TIF)

S4 Fig Electron density for dimer-dimer interfaces and active site nucleotides. (**A**) 2Fo–Fc electron density for α13 helix dimer-dimer interfaces observed in SAMHD1 tetramer structures. (Left) Interface I, present in SAMHD1(115–583)-ddGTP, SAMHD1(41–583)-ddGTP and pSAMHD1(115–626) tetramers. (Right) Interface II, present in SAMHD1(115–583)-ddGTP tetramer. Electron density, contoured at 1σ, is shown as blue mesh, the protein backbone is shown as a ribbon and sidechains in stick representation. (**B**) 2Fo–Fc electron density for the active site nucleotides in the SAMHD1(115–583)-ddGTP structure (Left) and SAMHD1(41–583)-ddGTP structure (Right). Electron density, contoured at 1σ, is shown as blue mesh, the ddGTP nucleotide and the metal co-ordinating residues shown as sticks, active site Fe is shown as a sphere (brown) and the protein backbone as a ribbon,
(TIF)

S5 Fig Active site configuration and identity of bound metal ions. (**A**) Anomalous-difference electron density map calculated from diffraction data recorded from a SAMHD1(115–583)-ddGTP crystal at the Fe absorption edge (1.735 Å). The region around the SAMHD1(115–583) active site is shown with the difference density, contoured at 5σ, shown as green mesh. The active site Fe is shown as a sphere (brown), the protein backbone as a ribbon, and the ddGTP nucleotide and surrounding residues shown as sticks. (**B**) X-ray fluorescence emission spectrum of a SAMHD1(115–583) crystal. Excitation energy was 12.66 keV. Peaks corresponding to Fe (K_α_ 6.40 keV and K_β_ 7.06 keV) and Zn (K_α_ 8.64 keV) emission energies are observed. A peak at 2.62 keV corresponding to the K_α_ emission from Cl is also present as the crystallisation solution contained 150 mM chloride ions. (**C**) ICP-MS analysis of transition metal ions contained in SAMHD1. Only Zn and Fe were detected above background. Results are displayed as the ratio of nmole metal per nmole of protein for each SAMHD1 construct tested, (n = 1). (**D**) Zn inhibition of SAMHD1 triphosphohydrolase activity. GTP stimulated SAMHD1(115–626) hydrolysis of dATP with and without addition of Zn^2+^ was analysed by IEX-HPLC. The plot shows the quantification of the time dependant depletion of substrate derived from integration of the dATP peak at each time point. Error bars represent the SEM from 3 independent experiments.
(TIF)

S6 Fig 2Fo–Fc electron density for allosteric site nucleotides. 2Fo–Fc electron density for allosteric site bound nucleotides in SAMHD1 crystal structures. The structure and associated bound nucleotide are indicated above. Electron density, contoured at 1σ, is shown as blue mesh and nucleotides are shown in stick representation.
(TIF)

S7 Fig *In vitro* phosphorylation of SAMHD1. (**A**) ESI-Q-TOF MS/MS fragmentation spectrum (m/z range 200 to 2000) the y_8_ and y_16_ molecular ions at m/z = 1004.55676 and 900.46710 unambiguously identify the tryptic peptide (N577-K596) and the phosphorylation at T592. (**B**) Mn^2+^ Phos-tag SDS-PAGE analysis of SAMHD1(115–626) pre (Lane 2) and post (Lane 3) treatment with CDK2/Cyclin A kinase. M, molecular weight markers.
(TIF)

S8 Fig ddNTP hydrolysis. SAMHD1 triphosphohydrolase activity against ddNTP substrates. GTP/dATP (0.2 mM ea.) stimulated SAMHD1(115–626) hydrolysis of the indicated ddNTP substrate (1 mM) was analysed by IEX-HPLC. The plot shows the quantification of the time dependant depletion of substrate derived from integration of the ddNTP peak at each time point. Error bars represent the SEM from 3 independent experiments.
(TIF)

S1 Table Primers used for SAMHD1 mutant synthesis. (PDF)

S2 Table Quantitative-PCR primers. (PDF)
