# Supplementary figures and images for: Phospho-dependent Regulation of SAMHD1 Oligomerisation Couples Catalysis and Restriction
Source: PLoS Pathog. 2015 Oct 2;11(10):e1005194. doi: 10.1371/journal.ppat.1005194 (PMC4592219; doi:10.1371/journal.ppat.1005194)

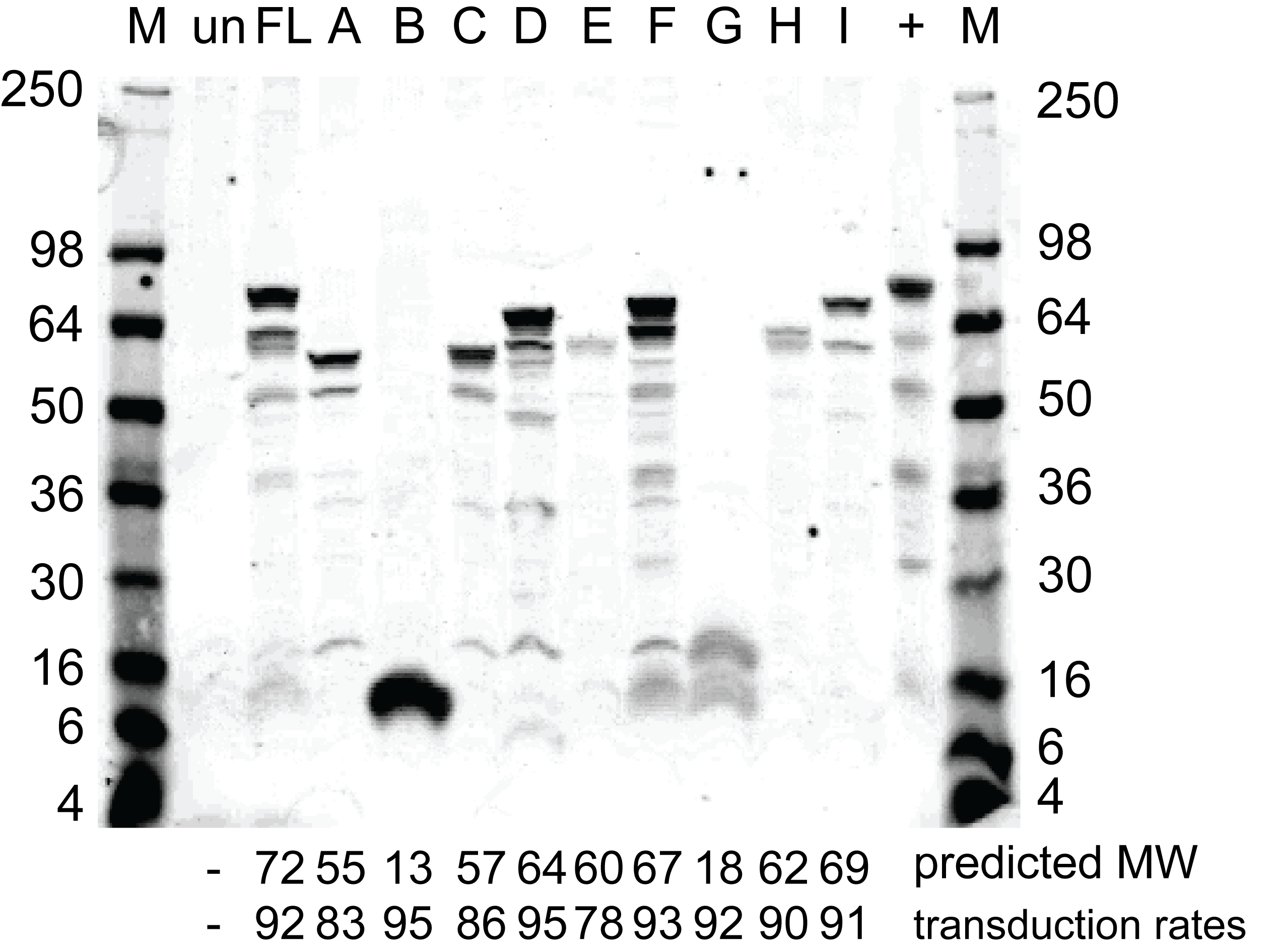

Supplement: S1 Fig — U937 cells transduced with SAMHD1 mutant viruses, labelled as in Fig 1A, were harvested at the time of infection with HIV-1GFP and SAMHD1 protein expression analysed in a 4–20% Tris-glycine gel followed by immunoblotting with anti-SAMHD1 antibody. M, marker; un, untransduced U937 cells; FL, full-length SAMHD1; +, SAMHD1-transfected 293T positive cell control. Marker molecular weights are indicated. Molecular weights for each mutant were predicted using the primary amino acid sequence in SeqBuilder (DNAStar) and are shown below along with the percentage YFP positive cells for each transduction. (TIF) [file ppat.1005194.s001.tif]

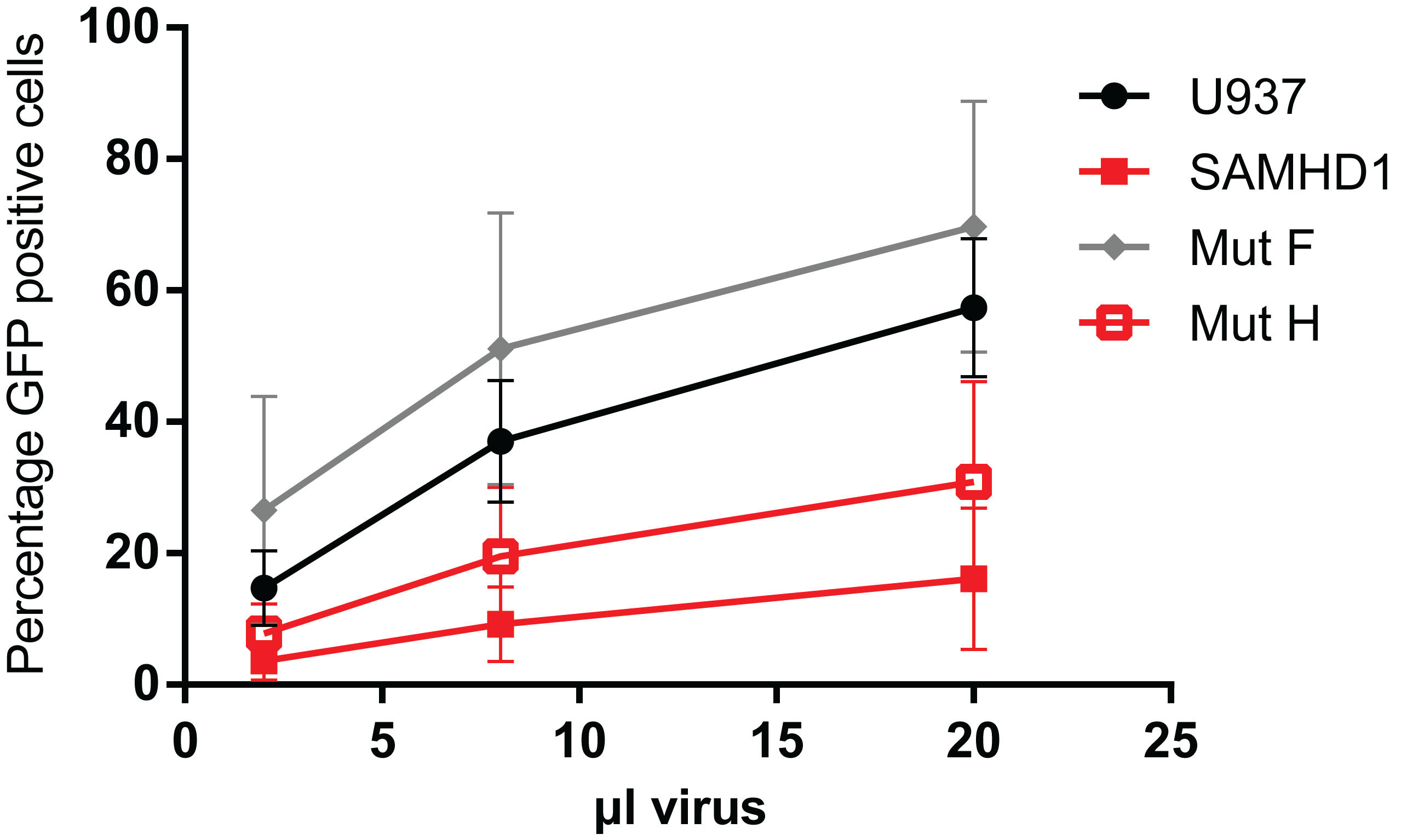

Supplement: S2 Fig — U937 cells (Parental) stably expressing either full-length SAMHD1, mutant F or mutant H were differentiated by adding PMA and 72 hours later infected with HIV-1GFP virus. The percentage of GFP positive cells was determined by flow cytometry and plotted relative to the amount of HIV-1GFP virus added to the cells. Error bars represent the range for n ≥7 independent experiments. (TIF) [file ppat.1005194.s002.tif]

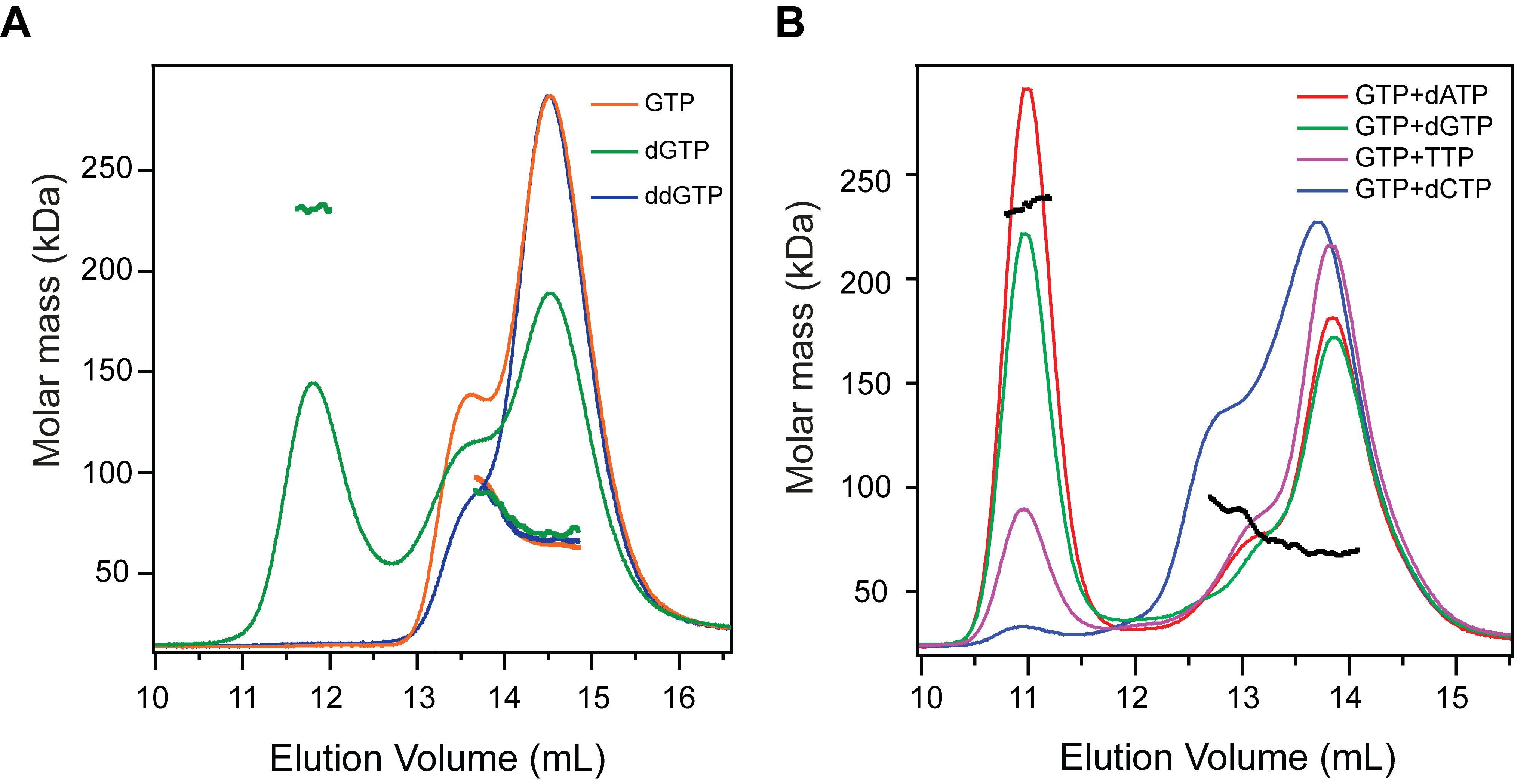

Supplement: S3 Fig — (A) SEC-MALLS analysis of monomer-dimer-tetramer equilibrium for SAMHD1(115–626) upon addition of 0.5 mM GTP (orange), dGTP (green) and ddGTP (blue). (B) SEC-MALLS analysis of monomer-dimer-tetramer equilibrium for SAMHD1(115–626) upon addition of 0.2 mM GTP and 0.5 mM each of dATP (red), dGTP (green), TTP (purple) and dCTP (blue). The chromatograms are the output from the differential refractometer and the displayed points are the weight-averaged molar masses determined at 1-second intervals throughout elution of chromatographic peaks. (C) SAMHD1 triphosphohydrolase activity against ddNTP substrates. GTP/dATP (0.2 mM ea.) stimulated SAMHD1(115–626) hydrolysis of the indicated ddNTP substrate (1 mM) was analysed by IEX-HPLC. The plot shows the quantification of the time dependant depletion of substrate derived from integration of the ddNTP peak at each time point. Error bars represent the SEM from 3 independent experiments. (TIF) [file ppat.1005194.s003.tif]

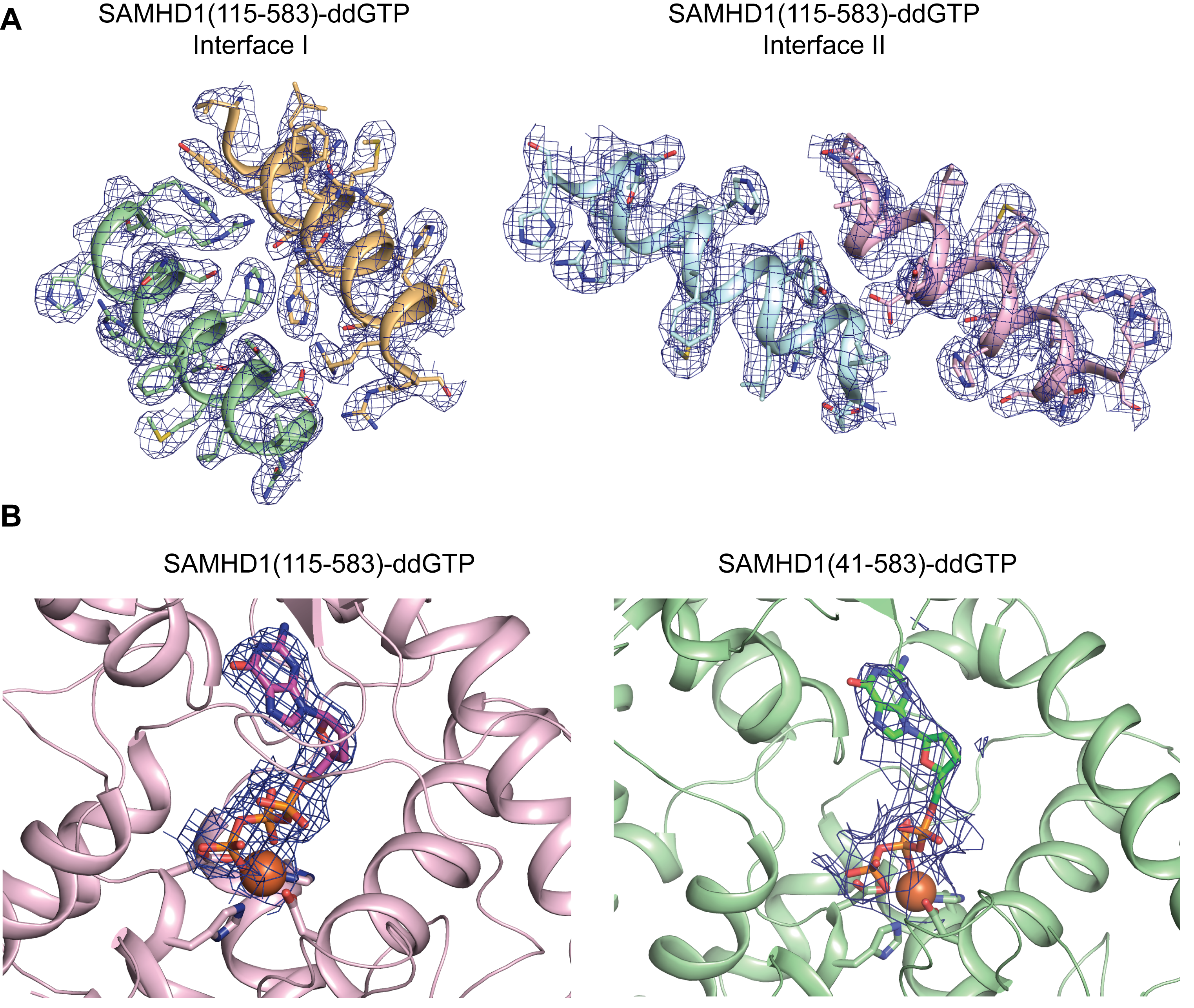

Supplement: S4 Fig — (A) 2Fo–Fc electron density for α13 helix dimer-dimer interfaces observed in SAMHD1 tetramer structures. (Left) Interface I, present in SAMHD1(115–583)-ddGTP, SAMHD1(41–583)-ddGTP and pSAMHD1(115–626) tetramers. (Right) Interface II, present in SAMHD1(115–583)-ddGTP tetramer. Electron density, contoured at 1σ, is shown as blue mesh, the protein backbone is shown as a ribbon and sidechains in stick representation. (B) 2Fo–Fc electron density for the active site nucleotides in the SAMHD1(115–583)-ddGTP structure (Left) and SAMHD1(41–583)-ddGTP structure (Right). Electron density, contoured at 1σ, is shown as blue mesh, the ddGTP nucleotide and the metal co-ordinating residues shown as sticks, active site Fe is shown as a sphere (brown) and the protein backbone as a ribbon, (TIF) [file ppat.1005194.s004.tif]

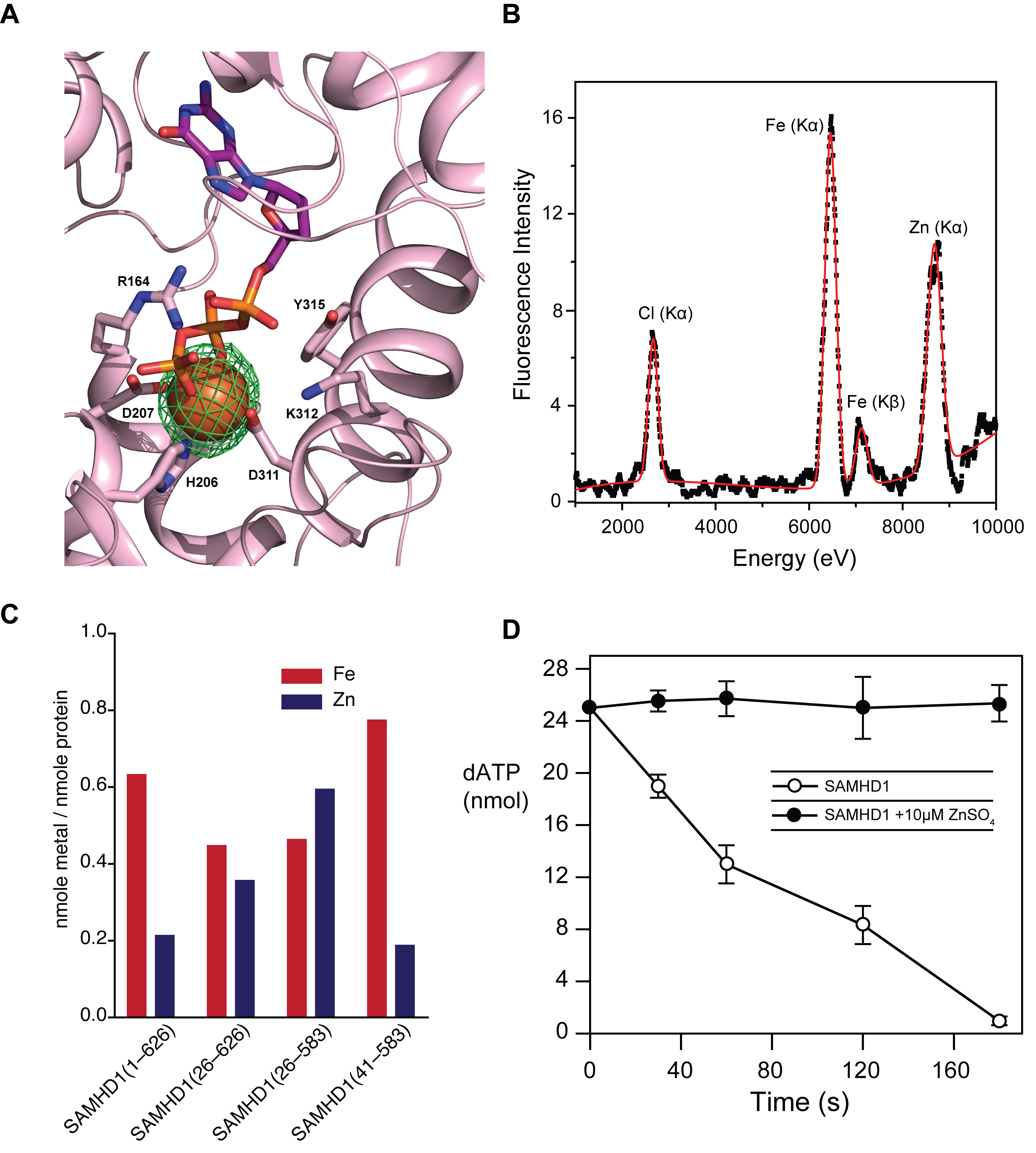

Supplement: S5 Fig — (A) Anomalous-difference electron density map calculated from diffraction data recorded from a SAMHD1(115–583)-ddGTP crystal at the Fe absorption edge (1.735 Å). The region around the SAMHD1(115–583) active site is shown with the difference density, contoured at 5σ, shown as green mesh. The active site Fe is shown as a sphere (brown), the protein backbone as a ribbon, and the ddGTP nucleotide and surrounding residues shown as sticks. (B) X-ray fluorescence emission spectrum of a SAMHD1(115–583) crystal. Excitation energy was 12.66 keV. Peaks corresponding to Fe (Kα 6.40 keV and Kβ 7.06 keV) and Zn (Kα 8.64 keV) emission energies are observed. A peak at 2.62 keV corresponding to the Kα emission from Cl is also present as the crystallisation solution contained 150 mM chloride ions. (C) ICP-MS analysis of transition metal ions contained in SAMHD1. Only Zn and Fe were detected above background. Results are displayed as the ratio of nmole metal per nmole of protein for each SAMHD1 construct tested, (n = 1). (D) Zn inhibition of SAMHD1 triphosphohydrolase activity. GTP stimulated SAMHD1(115–626) hydrolysis of dATP with and without addition of Zn2+ was analysed by IEX-HPLC. The plot shows the quantification of the time dependant depletion of substrate derived from integration of the dATP peak at each time point. Error bars represent the SEM from 3 independent experiments. (TIF) [file ppat.1005194.s005.tif]

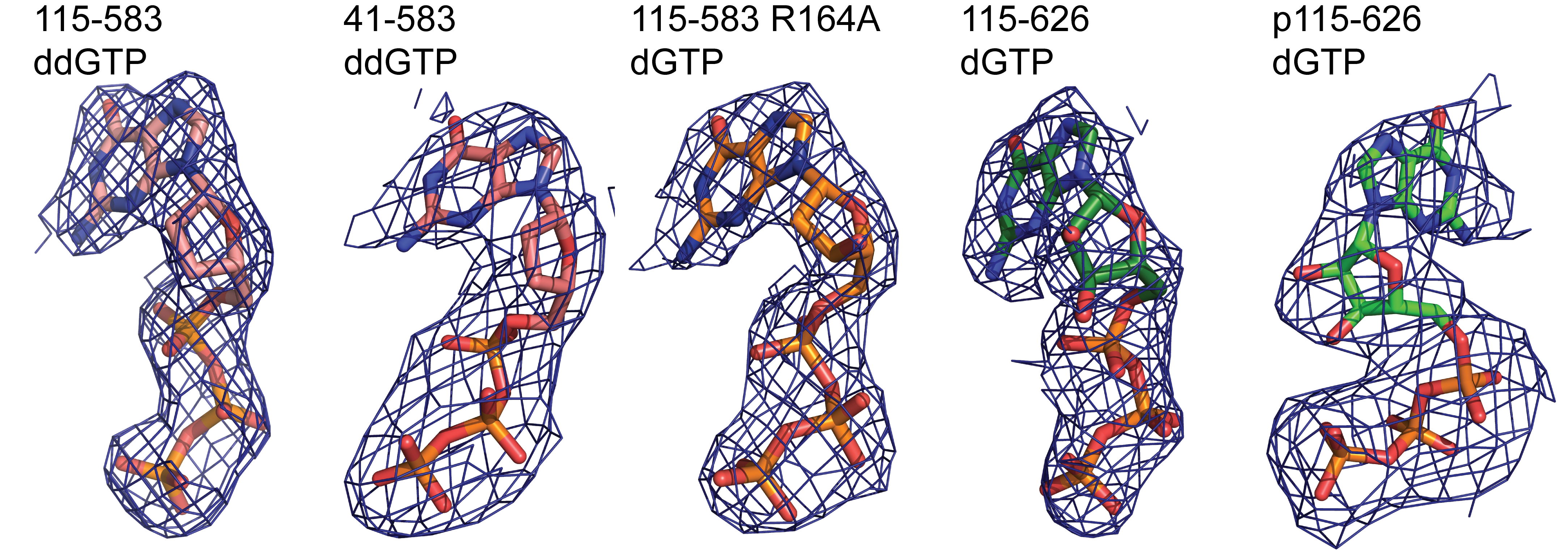

Supplement: S6 Fig — 2Fo–Fc electron density for allosteric site bound nucleotides in SAMHD1 crystal structures. The structure and associated bound nucleotide are indicated above. Electron density, contoured at 1σ, is shown as blue mesh and nucleotides are shown in stick representation. (TIF) [file ppat.1005194.s006.tif]

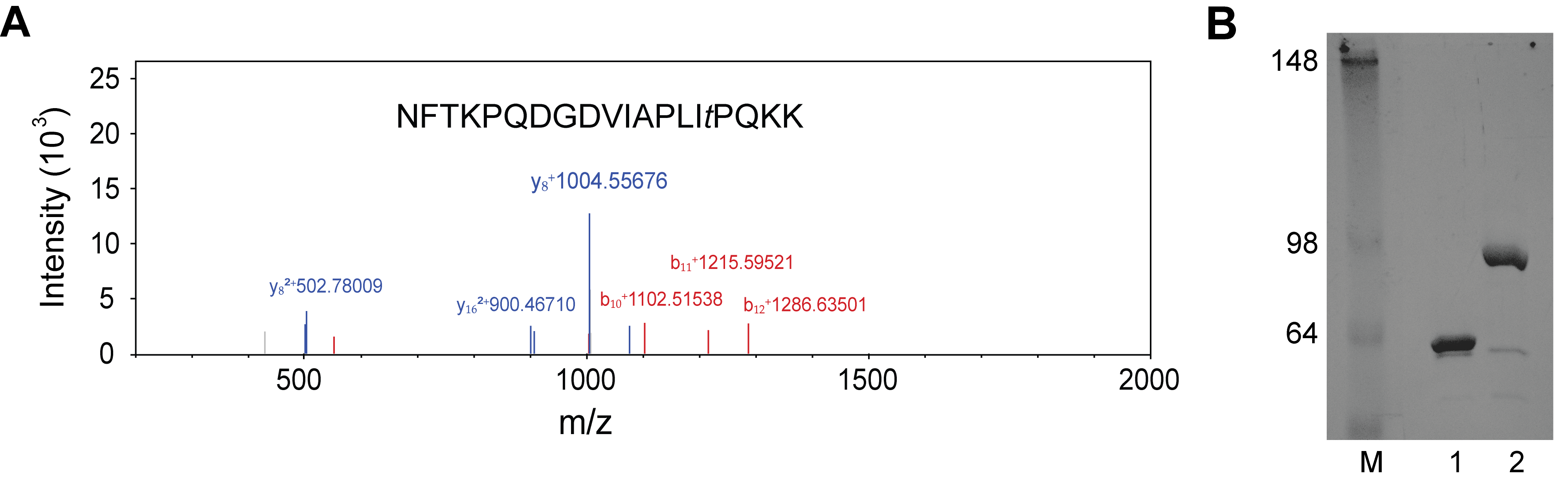

Supplement: S7 Fig — (A) ESI-Q-TOF MS/MS fragmentation spectrum (m/z range 200 to 2000) the y8 and y16 molecular ions at m/z = 1004.55676 and 900.46710 unambiguously identify the tryptic peptide (N577-K596) and the phosphorylation at T592. (B) Mn2+ Phos-tag SDS-PAGE analysis of SAMHD1(115–626) pre (Lane 2) and post (Lane 3) treatment with CDK2/Cyclin A kinase. M, molecular weight markers. (TIF) [file ppat.1005194.s007.tif]

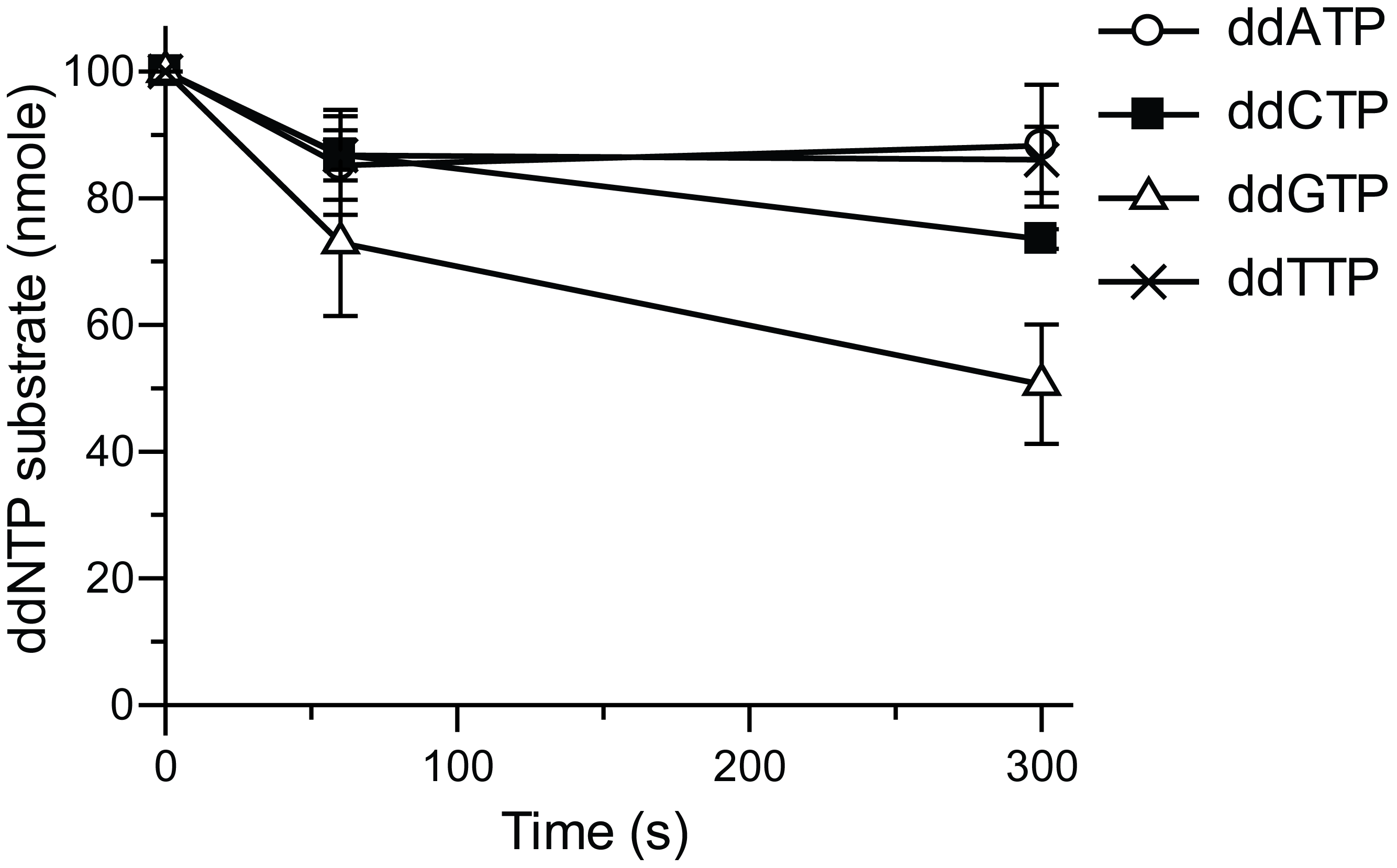

Supplement: S8 Fig — SAMHD1 triphosphohydrolase activity against ddNTP substrates. GTP/dATP (0.2 mM ea.) stimulated SAMHD1(115–626) hydrolysis of the indicated ddNTP substrate (1 mM) was analysed by IEX-HPLC. The plot shows the quantification of the time dependant depletion of substrate derived from integration of the ddNTP peak at each time point. Error bars represent the SEM from 3 independent experiments. (TIF) [file ppat.1005194.s008.tif]
