# Supplementary material for: Phospho-dependent Regulation of SAMHD1 Oligomerisation Couples Catalysis and Restriction
Source: PLoS Pathog. 2015 Oct 2;11(10):e1005194. doi: 10.1371/journal.ppat.1005194 (PMC4592219; doi:10.1371/journal.ppat.1005194)
Supplement: S1 Table — (PDF) [file ppat.1005194.s009.pdf]

S1 Table. Primers used for SAMHD1 mutant synthesis

Amplicon/Task	Forward primer (5´-3´)	Reverse primer (5´-3´)
SAMHD1 mutant A	caccATGGAAGTGATCAACGACCC	TCAGTCCTGAGGCTTGGTGAAGTTC
SAMHD1 mutant B	caccATGCAGCGGGCCGAC	TCAGGTGTCCACGTGAATCTGcac
SAMHD1 mutant C	GCCCCCGGACCCCTAGCAATACCCCTACCATGAAAGTGATCAACGACCCC	GGGGTCGTTGATCACTTTCATGGTAGGGGTATTGCTAGGGGTCCGGGGGC
SAMHD1 mutant D	caccATGCCTGGACTGGAACTGC	TCAGTCCTGAGGCTTGGTGAAGTTC
SAMHD1 mutant E	GTTTGTACAAAAAAGCAGGCTATGAAAGTGATCAAC	GGGTCGTTGATCACTTTCATAGCCTGCTTTTTTG
SAMHD1 mutant F	caccATGCAGCGGGCCGAC	TCAGTCCTGAGGCTTGGTGAAGTTC
SAMHD1 mutant G	GGTGCAGATTCACGTGGACACCGGCGACGTGATCGCCCCTCTGATCACCC	GGGTGATCAGAGGGGCGATCACGTCGCCGGTGTCCACGTGAATCTGCACC
SAMHD1 mutant H	GCCCCCGGACCCCTAGCAATACCCCTACCATGAAAGTGATCAACGACCCC	GGGGTCGTTGATCACTTTCATGGTAGGGGTATTGCTAGGGGTCCGGGGGC
SAMHD1 mutant I	caccATGCCTGGACTGGAACTGC	TCACATAGGGTCGTCCTTGAAC
Sequencing (YFP)	GTCTCTCCCCCTTGAACCTC	AGACGGCAATATGGTGGAAA
SAMHD1 (Puro)	GCTgttaacATGCAGCGGGC	GCTgttaacTCACATAGGGTCGTCCTT
SAMHD1 mutant F (Puro)	GCCTCAGGACTGAgaattccgcccc	ggggcggaattcTCAGTCCTGAGGC
Sequencing (Puro)	gtctctcccccttgaacctc	ccaaaagacggcaatatggt
