# Supplementary material for: Phospho-dependent Regulation of SAMHD1 Oligomerisation Couples Catalysis and Restriction
Source: PLoS Pathog. 2015 Oct 2;11(10):e1005194. doi: 10.1371/journal.ppat.1005194 (PMC4592219; doi:10.1371/journal.ppat.1005194)
Supplement: S2 Table — (PDF) [file ppat.1005194.s010.pdf]

S2 Table. Quantitative-PCR primers

Amplicon	Forward primer (5´-3´)	Reverse primer (5´-3´)	Probe (5´-3´)
Strong stop	TAACTAGGGAACCCACTGC	GCTAGAGATTTTCCACACTG	FAM-ACACAACAGACGGGCACACACTA-TAMRA
Second strand	CTGCGTCGAGAGAGCTCCTCTGGTT	TAACTAGGGAACCCACTGC	FAM-ACACAACAGACGGGCACACACTA-TAMRA
